# Controlling the Physical Properties in Hybrid Hydrogel Networks via Tunable Supramolecular Interactions

**DOI:** 10.1021/acs.macromol.5c00226

**Published:** 2025-06-03

**Authors:** Martin G.T.A. Rutten, Chiara Raffaelli, Maxime O. Grillaud, Riccardo Bellan, Wouter G. Ellenbroek, Patricia Y.W. Dankers

**Affiliations:** 1 Institute for Complex Molecular Systems, Eindhoven University of Technology, P.O. Box 513, Eindhoven 5600 MB, The Netherlands; 2 Department of Biomedical Engineering, Laboratory of Chemical Biology, Eindhoven University of Technology, P.O. Box 513, Eindhoven 5600 MB, The Netherlands; 3 Department of Applied Physics, Eindhoven University of Technology, P.O. Box 513, Eindhoven 5600 MB, The Netherlands; 4 Department of Chemical Engineering and Chemistry, Eindhoven University of Technology, P.O. Box 513, Eindhoven 5600 MB, The Netherlands

## Abstract

The complex interplay of covalent and noncovalent interactions is intrinsically connected to the formation of biological macromolecules, including proteins and carbohydrates. The design of synthetic materials that exhibit a similar interplay of such complex interactions is challenging. Most synthetic networks use purely covalent polymers in different concentrations to capture the bulk stiffness. Here, we combine covalent and dynamic network interactions in fully synthetic systems. In a systematic approach, permanent covalent cross-links are replaced by dynamic cross-links. Via this approach, network mechanics can be tuned over several orders of magnitude, i.e., from 10 to over 1000 Pa. This large tunability is achieved by changes in the molecular design of the dynamic cross-links, all while the fundamental design and concentration of the components are kept constant. Furthermore, where experiments showed a clear relationship between the design of the dynamic cross-links and the mechanical strength, coarse-grained molecular dynamics simulations showed a similar trend between networks mechanics and cross-link interaction strength. Overall, we show a new approach for the design of networks in which components and concentrations are kept similar, but a wide range of physical properties can be captured by tuning the molecular design of the cross-links. In this way, synthetic materials are brought closer to the design and tunability of biological matter.

## Graphical abstract

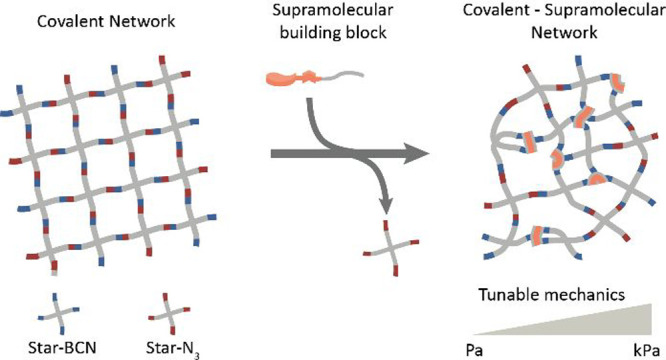

## Introduction

Natural tissue is composed of many different integrated structures, creating a complex mechanical and dynamic environment around cells, known as the extracellular matrix (ECM).^1–3^ The exact physical parameters can vary greatly depending on the tissue, e.g., soft brain and stiff bone.^4^ These differences originate from the molecular design of the used components and the combinations of these components via covalent and noncovalent interactions. Collagen type I and type IV form different networks based on variations in the amino acid sequence and the cross-links between the fibrillar structures.^5,6^ These cross-links can be either covalent in nature in terms of protein binding or noncovalent in the form of additional fibers or proteoglycans.^4,5^ This yields a large range of different physical parameters that can be tuned via molecular changes and interactions. The importance of these physical parameters has, over the years, been well-established using in-depth studies in which cell behavior was controlled using substrates with different stiffnesses, altering behavior such as spreading, migration, adhesion, and differentiation of stem cells.^4,7,8^ In addition, the local and macroscopic dynamics have recently been recognized as important parameters in terms of matrix remodeling via cellular forces.^3,9^

Many of the synthetic biomaterials that are currently used, have been developed around covalent polymers that can form hydrogel networks.^10–13^ In these systems, rather limited alterations in bulk stiffness are possible using different concentrations of the polymer, which also alters the structural properties and the network design.^14^ Furthermore, they lack the tunable dynamics that are found in nature. Dynamic behavior is often incorporated by the addition of reversible cross-links that can form or break via enzymes or external stimuli (e.g., pH, light, temperature, etc.).^15,16^ This in contrast to natural materials where dynamic behavior is inherently coupled with the molecular design of the monomeric building blocks. Changes in the molecular structure directly change the interaction strength between the components, which alters the macroscopic properties.^5,6^

Supramolecular hydrogels have recently been recognized as promising materials to mimic these dynamic aspects due to their noncovalent and reversible interactions.^13,17–19^ These systems can allow for tunable mechanics and dynamics by orthogonally tuning the concentration and the ratio of the supramolecular building blocks.^19,20^ Nevertheless, they are often based on one type of physical interaction, lacking the complex interplay of different physical interaction strengths combined with rigid covalent cross-links for more enduring mechanical stability over long time scales.

To overcome the limitations of pure covalent and pure supramolecular networks, double networks have been developed. Most of these double networks are chemically linked,^21,22^ while a few examples exist where a combination of noncovalent and covalent linkers is used.^23–29^ Albeit these double network hydrogels show promising results, the preparation procedures require multiple steps with toxic precursor materials and photopolymerization reactions. In addition, specific hydrogel compositions were made without investigating the role of each specific cross-link on material properties. For instance, these networks still rely on the crude methodology of changing macroscopic properties via concentrations, instead of nature’s sophisticated methods to control bulk properties via molecular design and interaction strength. Hence, the fundamental understanding of combining different types of cross-links in new multicomponent hydrogels is significantly lacking. Herein, this work aims to provide additional knowledge on this topic for the development of the next generation of hydrogels. Therefore, we create fully synthetic systems in which we use a combination of covalent and supramolecular cross-links to control macroscopic properties. We keep the global network components and concentrations of these components similar but alter the interaction strength between the cross-links to tune the physical properties over several orders of magnitude. In addition, to gain a more fundamental knowledge of the complex interplay between the different interactions, we combine our experimental results with coarse-grained molecular dynamics simulations.

The design of our hydrogels relies on a covalent backbone based on 4-arm-PEG_10k_ molecules (Figure 1A). Part of these molecules are end-functionalized with a BCN moiety, i.e., a strained cyclooctyne that can selectively react with azide moieties. In this respect, the other 4-arm-PEG_10k_ molecules are functionalized with an azide moiety (Figure 1A). Once dissolved, both 4-arm-PEG_10k_ molecules form transparent liquid solutions. Nevertheless, the use of these different molecules allows the formation of an elastic-like hydrogel upon mixing, i.e., the azide and BCN moieties react via a strain-promoted azide–alkyne click reaction (SPAAC), yielding a purely covalent network.^30,31^ Dynamic cross-links are introduced in these PEG networks via the addition of various supramolecular building blocks, based on ureido-pyrimidinone (UPy) molecules, in this case end-functionalized with azide-moieties (Figure 1B).^19,20,32–34^ UPy molecules can dimerize through self-complementary, quadruple hydrogen bonding. For the formation of larger hierarchical structures, the UPy moiety is coupled to a urea motif, facilitating lateral stacking through additional hydrogen bonding. Hydrophobic spacers can be used to shield the hydrogen bonding regions from the aqueous environment to increase the interaction strength between the monomers.

**Figure 1:**
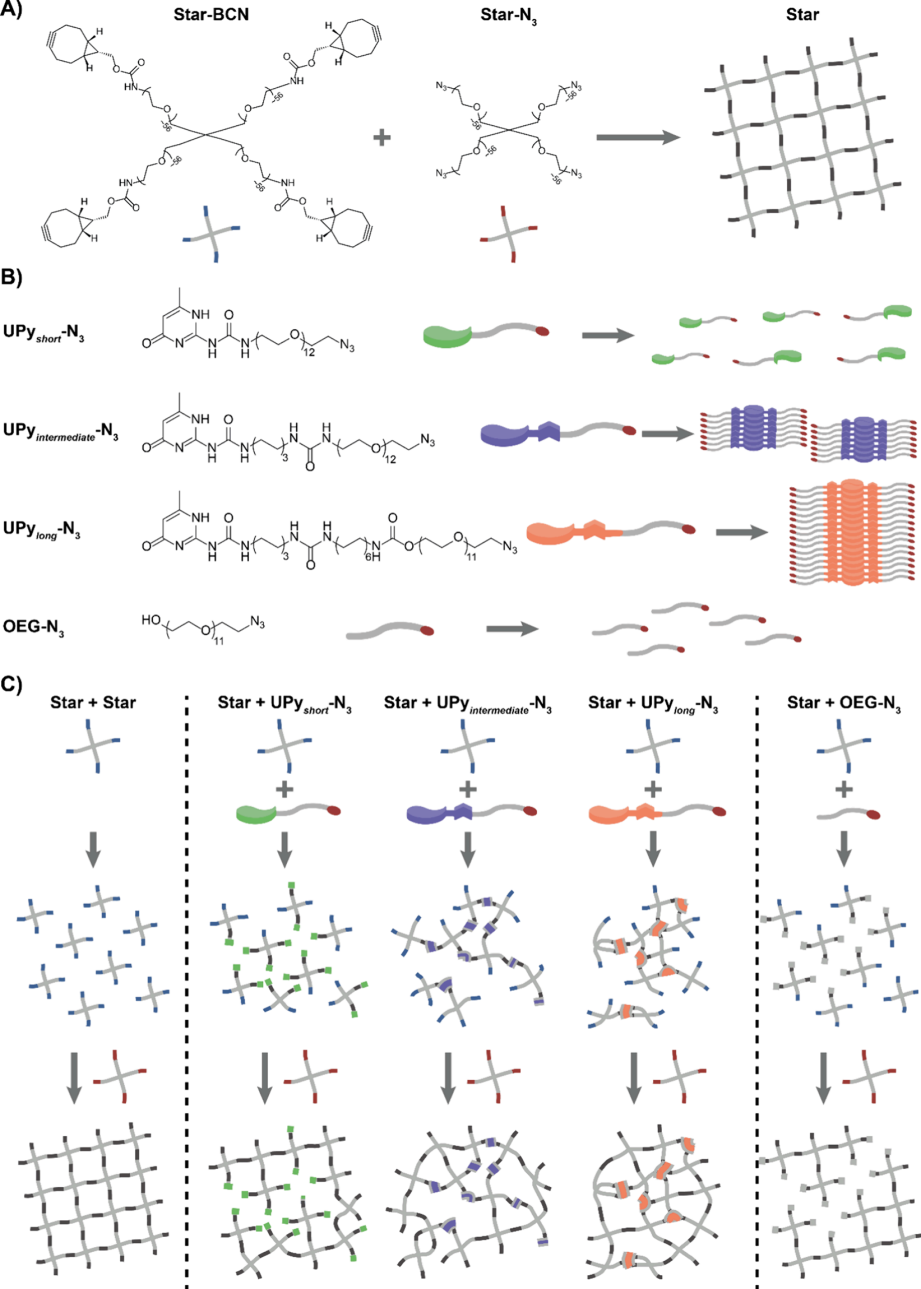
Design of the hydrogels employed, combining covalent cross-links with supramolecular interactions. (A) The core of the hydrogels is based on 4-arm-PEG_10k_ molecules end-functionalized with either BCN moieties or azide moieties. Upon mixing, an azide–alkyne click reaction results in the formation of a covalent network. (B) Supramolecular building blocks based on azide-functionalized ureidopyrimidinone molecules. Upon incorporation of urea moieties and hydrophobic spacers, the interaction strength between the molecules can be varied, which determines the length of the resulting fibers/clusters. **OEG-N**_**3**_ is used as a control. (C) **Star-BCN** is dissolved first and mixed with one of the UPy building blocks or **OEG-N**_**3**_ and allowed to react for 24 h. After 24 h, **Star-N**_**3**_ is added to the mixture to induce hydrogel formation.

Here, we use the molecular design of the UPy molecule to create different dynamic cross-links. More specifically, by changes in the molecular structure of UPy, the expected ability of the molecules to assemble into large structures is increased or reduced. In this respect, the UPy molecules form an ideal platform to investigate the effects of changed interaction strength due to the ability to specifically control the molecular design and, thereby, the supramolecular interactions. In addition, previously, a large effect was observed in network formation when UPy’s with a different interaction strength were used.^35^ The synthesized UPy molecules are here used to replace covalent cross-links in the star network, creating dynamic regions in a purely covalent backbone (Figure 1C). As the interaction between the UPy’s increases, they are able to yield larger supramolecular aggregates that are able to change the macroscopic properties in the ordered star-based PEG network. Important is that the concentrations of all components are kept similar, but due to molecular changes of the cross-links, large tunable effects in the macroscopic mechanics are obtained. Finally, we combine our findings with coarse-grained molecular dynamics simulations to bring more insight in these networks and guidance in terms of new network designs and possibilities.

## Materials and Methods

### Materials

All starting materials, chemicals, and solvents were obtained from commercial suppliers and used without purification. **OEG-N**_**3**_ was purchased from Polypure. **Star-N**_**3**_ was purchased from Jenkem (4arm-PEG10k-N_3_, MW ∼10 kDa). **Star-BCN** was synthesized by SymoChem according to reported procedures^36^ and stored in DCM with 1 mg of BHT as the stabilizer to prevent cross-linking. A detailed procedure for the synthesis of the various UPy’s is provided in the Supporting Information.

### Hydrogel Preparation

All compounds were dissolved separately before mixing. A small amount of **Star-BCN** was transferred into a small vial, and N_2_ flow was used to remove the DCM, after which the remaining product was dissolved in PBS for 30 min while being stirred. Each UPy compound or **OEG-N**_**3**_ was dissolved using a previous described protocol.^37^ In short, the weighted powder was dissolved in alkaline PBS for 30 min at 70 °C. Afterward, a small amount of HCl was added to neutralize the solution. A specific amount of UPy or **OEG-N**_**3**_ was added to bind 50% of all BCN moieties. To ensure complete functionalization, the mixture was stirred for 24 h. In case that no UPy compounds or **OEG-N**_**3**_ was added, **Star-BCN** was stirred for 24 h Next, **Star-N**_**3**_ was dissolved in PBS for 30 min while being stirred. A proper amount of **Star-N**_**3**_ was added to the BCN mixture to bind the remaining BCN moieties, reaching a final concentration of 3 wt %. After being mixed, the solution was immediately transferred into a rheometer to follow the gelation.

### Rheology

Rheological measurements were carried out on a TA Instruments Dynamic Hybrid Rheometer 3 in a 20 mm aluminum cone–plate (2.007°) geometry with a truncation gap of 56 μm. A solvent trap was used to minimize sample drying. Samples were loaded at 37 °C and allowed to equilibrate for a short period. Hydrogel formation was followed, until a plateau was reached, by measuring the complex modulus (G*) via an oscillating deformation of amplitude γ = 0.01 at a frequency ω = 1 rad/s. Frequency dependence was measured from ω = 0.01 to 100 rad s^–1^ at a strain amplitude of γ = 0.01. Stress-relaxation measurements were preceded by a time sweep with low strain amplitude and frequency (γ = 0.005, ω = 0.5 rad/s) before applying a strain of γ = 0.1 (strain rise time: 0.05s) and monitoring the stress for 10,000 s. The data were normalized using the stress obtained after 1 s as the starting point.

### Molecular Dynamics Simulations for the Relaxation Modulus

Mimicking the experimental setup in a coarse-grained molecular dynamics bead–spring simulation model, each star was represented via 4 arms of 28 beads each and 1 center bead, with each bead representing 2 PEG repeat units. Attaching a supramolecular moiety to a **Star-BCN** arm was done prior to simulations by assigning UPy molecules randomly and independently to the arms of the **Star-BCN** molecules with a probability equal to the desired UPy-fraction, which is 50% in most of our experiments (Figure 2). Once the simulation is started, reversible bonds are formed between the UPy molecules, and covalent bonds are formed between the BCN and azide moieties. The ratio of these partially-UPy-functionalized **Star-BCNs** to **Star-N**_**3**_ is such that the azide–alkyne reaction happens stoichiometrically. 150 star polymers are put in a simulation box with sizes to match 3 wt % gel. Simulations are performed using LAMMPS^38^ using the bond/create fix for the click reaction. The interaction between the reversible cross-links is based on the standard Lennard-Jones interaction with varying interaction strength ε and interaction distance σ between UPy beads with distance *r*_*ij*_:

$$u\left(r_{i,j}\right)=4\varepsilon \left[{\left(\frac{\sigma }{r_{ij}}\right)}^{12}-{\left(\frac{\sigma }{r_{ij}}\right)}^{6}\right]$$

**Figure 2:**
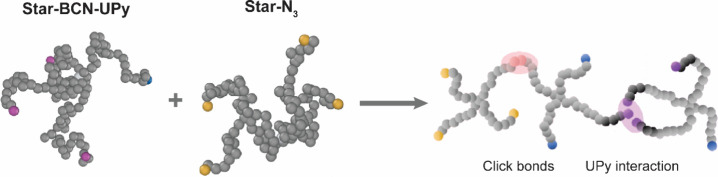
Design of simulation experiments corresponding to the experimental hydrogels. Stars are represented as beads, with inert beads in gray, −N_3_ moieties in yellow, −BCN moieties in blue, and −UPy moieties in purple. For simulations, these UPy moieties are all linked to BCN groups before any other molecules are added. Subsequently, **Star-N**_**3**_ is added to induce the formation of covalent bonds between BCN and azide groups, while UPy groups cluster in reversible bonds.

This choice allows our model to capture the effect of larger clusters of reversible linkers on the network mechanics. The beads representing the PEG chains interact purely repulsively to capture the good-solvent behavior via the Weeks–Chandler–Anderson potential (WCA):

$$u\left(r_{ij}\right)=\{\underset{0}{4\varepsilon_{WCA}\left[{\left(\frac{\sigma }{r_{ij}}\right)}^{12}-{\left(\frac{\sigma }{r_{ij}}\right)}^{6}\right]+\varepsilon_{WCA}}\begin{array}{c} ifr\leq 2^{1/6}\sigma \\ ifr\geq 2^{1/6}\sigma \end{array}$$

The beads are connected into chains using harmonic springs with a rest length of 1 (the unit of length in our simulations). We use σ = 1.3 to prevent chain crossings and vary the binding energy ε between 0 and 3.0 to mimic the variations of the UPy binding strength in the experiments, yielding different degrees of clustering. The reaction is not based on any attractive potential between the azide and BCN moieties but solely on the moieties finding each other through thermal motion. After the azide–alkyne click reaction, both beads become inactive and form inert beads. The mass of the polymer beads is set to *m* = 1, the bond length is set to *b* = 1, and the WCA length scale is set to σ = 1.3. This fixes our unit of time as $t=b\sqrt{m/\varepsilon_{WCA}}$ . Gelation and rheological/relaxation simulations are performed with an integration time step of 0.004.

### Stress Relaxation Moduli from Simulations

Stress relaxation measurements were simulated by displacing the upper surface of the simulation box to a new shape and thereby following the change in shear stress and comparing it to the shear stress in a simulation with no strain ( $\overset{®}{\sigma_{xy,0}\;}$ ), resulting in the relaxation modulus:

$$G\left(t\right)=\frac{\sigma_{xy,0}\left(t\right)-\overset{®}{\sigma_{xy,0}\;}}{\gamma }$$

The long-time behavior of the relaxation modulus distinguishes fluid behavior (*G*(*t*) approaches zero for long times) from solid behavior (*G*(*t*) approaches the equilibrium shear modulus for long times). We compare the resulting equilibrium shear modulus to an analytic model in which we estimate the number of polymer strands in the network and use Kuhn theory^39^ to obtain a prediction for the equilibrium modulus in terms of the thermal energy *k*_B_ *T* times the number density of polymer strands *N*_strand_/*V*:

$$G\approx \frac{N_{\mathrm{strand}}}{V}k_{B}T$$

## Results and Discussion

### Design of Hydrogel Components

The main components of the used networks are **Star-BCN** and **Star-N**_**3**_, which upon mixing form covalent cross-links to yield a viscoelastic hydrogel network. A concentration of 3 wt % was used to ensure the formation of a relative stiff hydrogel, i.e., *G*′ > 1000 Pa.^40^ The obtained *G*′ for this pure covalent network is in addition accessible by UPy hydrogels by itself, ensuring that incorporation of UPy molecules could alter the bulk properties in these hybrid networks.^19,20,34,37,41,42^ To incorporate dynamic regions, azide-functionalized UPy molecules are incorporated in a ratio to bind half of the BCN moieties of **Star-BCN**, leaving the remaining half of the BCN moieties available to form covalent links with **Star-N**_**3**_ molecules (Table S1). UPy molecules have previously been shown to be able to dimerize via 4-fold hydrogen bonds in organic solvent.^32^ In aqueous solutions, however, these hydrogen bonds compete with the solvent, meaning that once dissolved, we expect the presence of mere monomers for UPy-OEG_12_-N_3_ (Figure 1B), named **UPy**_*short*_ **-N**_**3**_ afterward. As mentioned before, to increase the expected size of UPy assemblies, these hydrogen bonds can be shielded from the aqueous environment with hydrophobic alkyl spacers, while urea groups can be incorporated to form additional hydrogen bonds.^19,20,37^ In this regard, two different UPy molecules are used. First, only one hydrophobic spacer is introduced, i.e., UPy-C_6_-U-OEG_12_-N_3_ (**UPy**_*intermediate*_ **-N**_**3**_), shielding only the UPy core from the surrounding solvent (Figure 1B). Alternatively, a third UPy molecule is used with an additional hydrophobic spacer to also shield the urea motive from the aqueous environment, i.e., UPy-C_6_-U-C_12_-U-OEG_11_-N_3_ (**UPy**_*long*_ **-N**_**3**_). Previously, it was observed that the effect of these hydrophobic spacers is significant in formation of higher order structures.^35^ That is, the absence of hydrophobic spacers results in the formation of significant shorter aggregates, and thus, **UPy**_*intermediate*_ **-N**_**3**_ is expected to form only short fibers, while **UPy**_*short*_ **-N**_**3**_ will likely be unable to form aggregates.^35^ As a control for the latter, OEG_11_-N_3_ (**OEG-N**_**3**_) is included to ensure the formation of monomeric ends around **Star-BCN** (Figure 1B).

### Formulation of Various Dynamic Hydrogels

Hydrogels were formed by mixing azide-functionalized UPy’s with **Star-BCN’s** in a ratio to ensure half of all BCN moieties could react with an UPy (Table S1). Therefore, BCN was first dissolved in pure PBS, while the various UPy compounds and **OEG-N**_**3**_ were dissolved in alkaline solution and afterward neutralized.^37^ A specific amount of UPy or **OEG-N**_**3**_ was then added to bind 50% of all of the BCN moieties. After 24 h, to ensure complete incorporation of the supramolecular components, **Star-N**_**3**_ was added to form covalent click bonds between the PEG stars. Overall, this approach resulted in **Star-BCN’s** in which 50% of the arms was attached to a **Star-N**_**3**_ and 50% to a UPy molecule. This resulted in a complex network of covalent cross-links between azide and BCN moieties and supramolecular cross-links between the UPy molecules attached to the stars. To change the physical properties of the hydrogel network, the molecular structure of the UPy cross-links was varied via the previously mentioned hydrogen bonding units and hydrophobic spacers (Figure 1C). These alterations were expected to yield different degrees of clustering compared to the UPy molecules.

### Formation of Hydrogel Networks

To follow the formation of the hydrogel networks, small amplitude oscillatory rheology was performed over time (Figure 3A). Formation of elastic-like networks only started once **Star-N**_**3**_ was added to UPy functionalized **Star-BCNs**, which were liquid solutions before. Even when **UPy**_**long**_ **-N**_**3**_ was used, which was expected to form long and rigid fibers, the UPy molecules with **Star-BCN** were unable to create a percolating network without the presence of the **Star-N**_**3**_. After the addition of **Star-N**_**3**_, a clear relationship between the used UPy molecule and formation time is observed. For the purely covalent network, an elastic-like (*G*′ > *G*″) hydrogel starts to form minutes after mixing of the two stars, reaching its maximum value of *G*′ just under 2 h (Figure 3A, B and Figure S18), in accordance with previous results from star PEG hydrogels.^40^ This is in large contrast to especially the incorporation of **UPy**_*short*_ **-N**_**3**_, which starts to form an elastic-like hydrogel after ∼1 h and which took ∼15 h before reaching its maximum *G*′ value. In addition, its formation over time is very similar to networks with **OEG-N**_**3**_ (Figure S18), which also starts to form after 1 h and which takes several hours to reach its maximum stiffness. These results indicate that the absence of hydrophobic spacers in **UPy**_*short*_ **-N**_**3**_ prevents the formation of clusters and fibers, yielding free dangling ends around **Star-BCN**, similar to incorporation of **OEG-N**_**3**_. Nevertheless, the difference in time before the maximum stiffness is reached (Figure 3B) could indicate toward some interactions between UPy end-groups around the star, which contribute marginally to the final stiffness over time.

**Figure 3:**
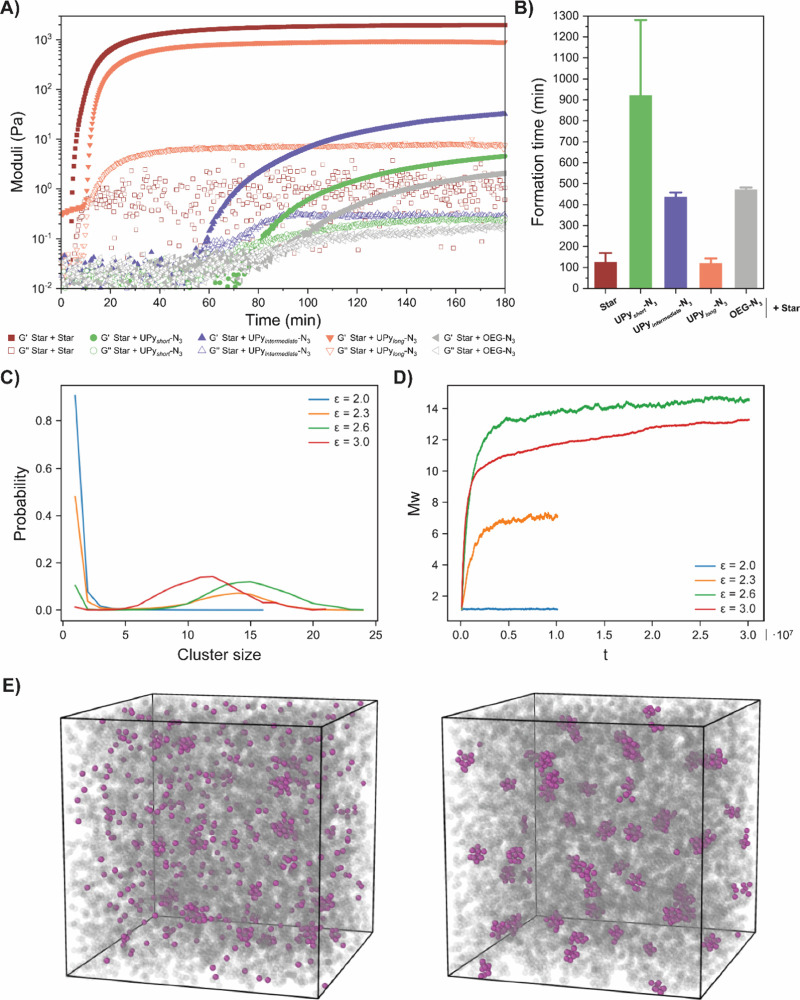
Various UPy molecules have varying degrees of clustering. (A) Initial formation of the various hydrogel networks. The pure covalent network forms relatively fast. Incorporation of UPy’s with weak interaction increases the needed formation time. The networks remain liquid solutions much longer when the interaction decreases. (B) Time required before the maximum storage modulus is obtained for the various networks, showing a clear trend between formation time and the used UPy’s. (C) Cluster distribution of R-cluster size at equilibrium for various interaction strengths (ε = 2.0, 2.3, 2.6, and 3.0), showing a clear distinction between a peak of clustered beads (size >4) and unbound beads. When Lennard-Jones potential increases, the clusters and the number of beads in a cluster become larger. (D) Weight-average clusters over time for various interaction strengths (ε = 2.0, 2.3, 2.6, and 3.0). When interaction strength between the UPy’s increases, it takes longer to reach equilibrium as clusters keep growing. (E) Snapshots to show morphological changes in the network when different interaction strengths are used for the UPy’s. Two simulation boxes are shown, one with weak interaction (ε = 2.0) between the UPy’s (left) and one with strong interaction (ε = 3.0) between the UPy’s (right), showing varying degrees of clustering.

Upon increased UPy interaction strength, i.e., **UPy**_*intermediate*_ **-N**_**3**_ and **UPy**_*long*_ **-N**_**3**_, the formation time decreased, where incorporation of the latter one results in formation times similar to the pure covalent network (i.e., a network of only **Star-N**_**3**_ and **Star-BCN** in which all BCN and azide moieties form a covalent bond) (Figure 3B). These results indicate that incorporation of larger UPy clusters decreases the time needed to reach a stable bulk stiffness.

Regarding the MD simulations, insight is provided about the size of dynamic clusters as a function of their interaction strength (Figure 3C). Once simulations are started, dynamic cross-links start to form. For a weak interaction strength (ε), only small clusters were observed over time. An increase in the interaction strength also led to an increase in the typical cluster size (Figure 3C). Simultaneously, the time before clusters reach their final size increases when the interaction strength is stronger (Figure 3D). After the formation time, once hydrogels are formed, the effect of different interaction strengths is also clearly observed once snapshots are taken of the networks, clearly showing the difference between the different cluster sizes in the network upon increased and decreased interaction strengths (Figure 3E).

In this case, the simulations and macroscopic rheology bring insight into the complex interplay between the macroscopic network properties and the microscopic cluster formation. The simulations showed that by increasing the interaction strength, the formation of clusters requires more time. This is explained by the tendency of clusters to keep growing into larger aggregates, after which these can further cluster together before finally reaching an equilibrium. Larger aggregates take much longer to assemble than smaller ones, in large part due to the fact that during assembly, the system is being depleted of unbound moieties, lowering their concentration that progressively increases the time between binding events. This effect also depends on the amount of UPy’s used in the network (Figure S19). On a macroscopic level, rheology showed that the use of long UPy cross-links, i.e., **UPy**_*long*_ **-N**_**3**_, leads to a faster formation of the hydrogel network. For a weak interaction strength, the simulations showed that cluster formation time is much shorter, as the clusters do not keep growing and remain relatively small. Once shorter UPy’s were used in the rheology, a longer macroscopic network formation time was observed. This longer formation time when **UPy**_*short*_ **-N**_**3**_ and **UPy**_*intermediate*_ **-N**_**3**_ were used is attributed to two effects: First, the smaller UPy clusters are unable to act as effective cross-links as there is only a small number of cross-links per aggregate that can link different stars together. Second, these small aggregates can effectively withdraw **Star-BCNs** from the overall network, as a small UPy cluster can bind to several free BCN moieties of one star, without forming cross-links to other stars, lowering the number of cross-links and molecules that can effectively carry load in the network. This is in contrast to the large UPy clusters, which are composed of many UPy molecules, connecting multiple stars.

### Tunable Viscoelastic Behavior

The observed differences in network formation are expected to result in different elastic and viscous properties once they are incorporated into the covalent star network. Rheological experiments after network formation showed a large effect in the final moduli (Figure 4A, B). Not surprisingly, the purely covalent star network reached the highest *G*′ of more than 2 kPa. Incorporation of the shorter UPy molecules reduced the storage modulus of the networks significantly, especially **UPy**_*short*_ **-N**_**3**_ yielded a network that is more than 200 times softer in terms of the storage modulus, i.e., ∼10 Pa. For comparison, previously, to lower the stiffness of star PEG hydrogels to ∼10 Pa, the concentration had to be lowered more than 3 times, while here, the concentration is kept constant.^40^ The obtained stiffness when **UPy**_*short*_ **-N**_**3**_ was used is in addition similar to the *G*′ obtained when **OEG-N**_**3**_ is used, confirming the hypothesis that the absence of hydrophobic spacers in **UPy**_*short*_ **-N**_**3**_ prevents formation of clusters and fibers, yielding free dangling ends around **Star-BCN**, similar to incorporation of **OEG-N**_**3**_.

**Figure 4:**
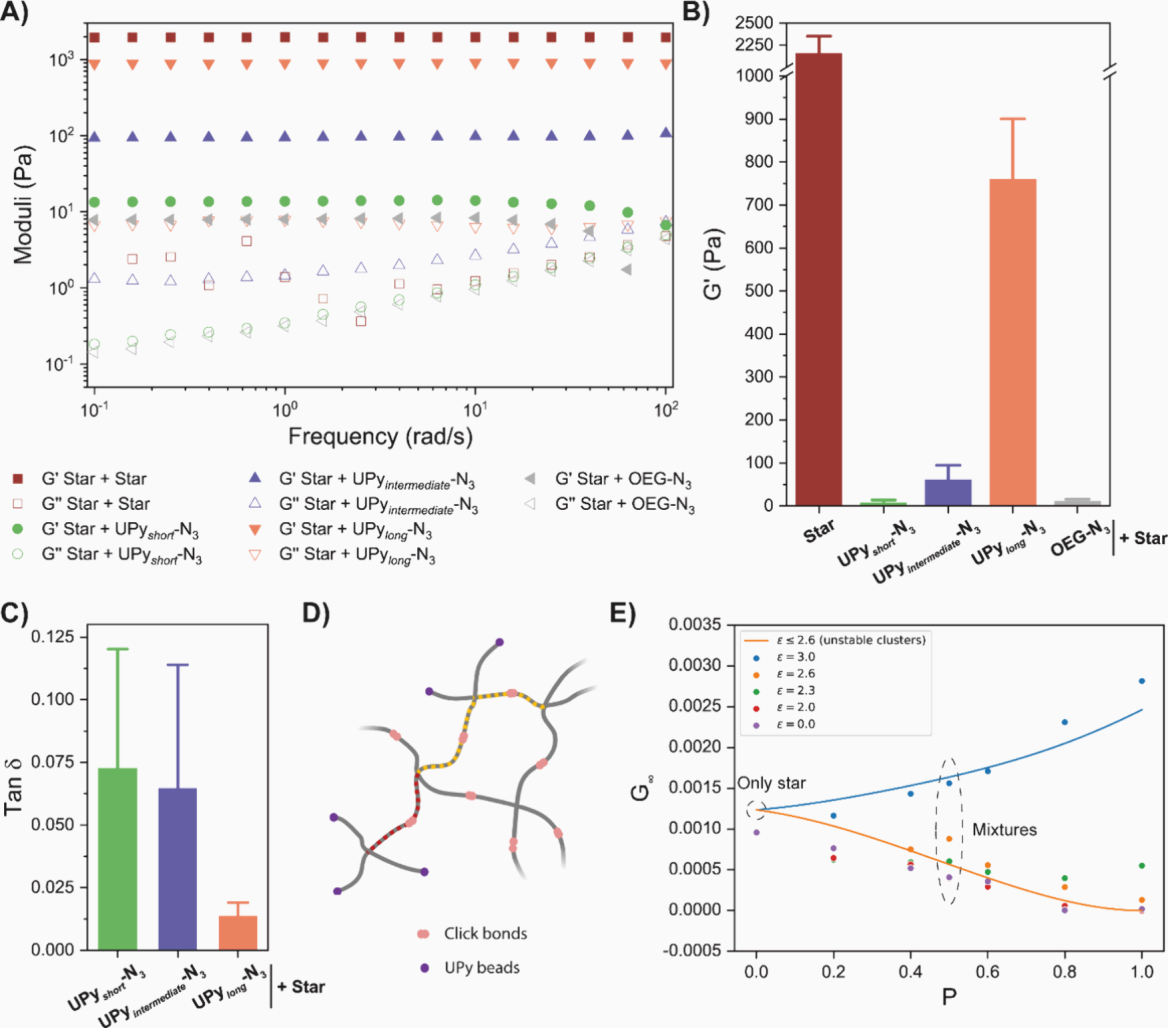
Mechanical properties of the various networks with different UPy’s. (A) Experimental frequency measurements, which show the time-dependent mechanical behavior of the different networks (*y* = 0.01). To study the effect of the different UPy’s, in all cases, 50% of **Star-N**_**3**_ is replaced with the corresponding UPy. All networks form elastic-like viscoelastic materials, but with differences in the absolute values of the moduli. (B) Final storage moduli of the various networks, showing that incorporation of UPy’s with weaker interaction decreases the modulus of the network. (C) Effect of the various UPy’s on the viscoelastic properties of the hydrogels, upon increasing the interaction strength, the viscous modulus of the network rises less than the elastic modulus, thereby lowering the loss tangent. (D) Schematic illustration of how we deal with dangling ends when we make our theoretical predictions for the moduli. While usually, in Kuhn theory, every covalent strand contributes to the modulus, the red-dashed strand here only connects three dangling ends (with UPy’s) to the network. When the UPy-bonds are weak, those will not contribute. Similarly, we count the yellow-dashed pair of covalently bonded strands as a single strand because the other two arms of the star in its middle are dangling as well. (E) Theoretical prediction of the modulus based on estimating the number of strands (solid lines), together with the moduli obtained from the simulations (dots). The interaction strength of the UPy’s as well as the amount of UPy’s can influence the modulus of the network.

Once longer UPy molecules were used, i.e., **UPy**_*intermediate*_ **-N**_**3**_ and especially **UPy**_*long*_ **-N**_**3**_, the storage modulus of the network increased again to ∼100 Pa and almost ∼1000 Pa, respectively. This increase in stiffness shows that a stronger interaction between the UPy molecules results in significant alterations in the macroscopic properties of the overall network. The supramolecular interactions between the molecules are significantly important to alter the bulk stiffness of the star-based PEG network.

Interestingly, upon probing various time scales via frequency measurements, even the softest network remained an elastic-like network (*G*′ > *G*″), without any indication of liquifying below 0.1 rad s^–1^ (Figure 4A). These stable *G*′ and G″ curves indicate the ability of these networks to form stable hydrogels over long time-scales while tuning the storage modulus over several orders of magnitude. Extraordinarily, this extreme tunability is possible without changing the concentrations of the components. Additionally, while all hydrogels are clearly elastic over the measured time scales, incorporation of the longer UPy molecules results in a small decrease of the viscous to elastic ratio, expressed by tan δ = *G*″/*G*′, which decreased from ∼0.075 to ∼0.012 (Figure 4C).

To analyze the mechanical properties of gels in the simulations, a predictive model based on the standard rubber elasticity was developed. Important is the molecular picture of the networks, as two types of strands can be formed around the star centers: the covalent bond between **Star-BCN** with **Star-N**_**3**_ and dynamic clusters connected to a **Star-BCN**. The strength of the second type of strand varies as it depends on the interaction strength of the dynamic molecules. A high interaction strength results in the formation of stable clusters that provide a stable mechanical strand. In contrast, when weak clusters are formed, they do not contribute to the long-term mechanical stiffness. In addition, the weak clusters also lower the effectiveness of the covalent strands in carrying loads through the presence of dangling ends (Figure 4D).

Simulations showed that upon lowering the interaction strength below ε = 2.6, a gradual decrease in the predicted modulus is obtained (Figure 4E). When comparing this simulation data to the experimental data, the relatively large decrease in *G*′ for **UPy**_*short*_ **-N**_**3**_ and **OEG-N**_**3**_ that was observed in the experimental data is somewhat less pronounced in the simulation data, although in both cases a decrease is observed. The difference between experiments and simulations is probably due to noise in the simulation that affects the fits needed to obtain the moduli. Additionally, this difference might highlight a small discrepancy between the experimental data and the simulated model. Whereas the synthesized UPy’s form long elongated clusters, the clusters in the model are roughly spherical in shape. Furthermore, as mentioned previously, the simulations and experimental rheology probe different length scales, where the latter one is much larger. This could also explain part of the difference between simulations and experimental data, for example, if the experimental gel samples contain inhomogeneities on length scales that are larger than those probed in simulations. Nevertheless, in correspondence with the experimental data, the modulated difference between cross-links without any interaction strength (ε = 0) or a very weak interaction strength (ε = 2.0) is rather small, similar to the experimental *G*′ for **OEG-N**_**3**_ and **UPy**_*short*_ **-N**_**3**_. These results confirm the hypothesis that **UPy**_*short*_ **-N**_**3**_ will mainly form individual monomers. Furthermore, when the interaction strength is increased in the simulations, ε = 2.3 and 2.6, an increase in the modulus can be observed, although never surpassing the initial covalent modulus, as we also see in the experiments. When **UPy**_*intermediate*_ **-N**_**3**_ and especially **UPy**_*long*_ **-N**_**3**_ are incorporated, a large increase in the storage modulus is observed, but never reaching the stiffness of the pure covalent network (Figure 4A, B). Interestingly, simulations show that by increasing the interaction strength beyond ε = 2.6, to ε = 3.0, an increase in the moduli beyond the initial stiffness of the pure covalent network can be achieved, even when large strains are applied (Figure S20). This highlights the possibilities that could arise with the design of alternative UPy molecules with stronger interaction strength.

Next, we focused in more depth on **UPy**_*long*_ **-N**_**3**_, as these molecules can significantly increase the storage modulus when compared to those of **UPy**_*short*_ **-N**_**3**_ and **UPy**_*intermediate*_ **-N**_**3**_. Additional experiments were conducted, in which varying amounts of **UPy**_*long*_ **-N**_**3**_ were used to tune mechanical properties (Table S2, Figure S21). Instead of functionalizing 50% of all BCN arms with a UPy molecule, the amount of **UPy**_*long*_ **-N**_**3**_ was varied to bind 20%, 40%, and 60% of all BCN moieties. In contrast to the large variations measured in storage modulus for the different UPy’s, only marginal differences are observed when changing the amount of **UPy**_*long*_ **-N**_**3**_ (Figure S21). These small differences are also in accordance with the simulations, where differences in moduli are rather small when changing the amount of strong interacting clusters, e.g., when incorporating different amounts of ε = 2.6 clusters (Figure 4E). Instead of changes in *G*′, a small effect of more **UPy**_*long*_ **-N**_**3**_ incorporation, could potentially be observed in the tan δ (Figure S21), which increased slightly from ∼0.02 to ∼0.03 upon incorporation of more **UPy**_*long*_ **-N**_**3**_. Nevertheless, differences are rather small, especially compared to the differences between using different UPy molecules (Figure 4C).

### Dynamic UPy’s Affecting the Bulk Dynamics of the Network

Finally, in addition to probing the time-dependent viscoelastic behavior via frequency measurements, stress relaxation experiments were conducted, which followed the decrease in stress after a strain was applied (Figure 5A). Compared to the large differences in storage moduli, only small changes in the time-dependent stress decay were observed, as incorporation of shorter UPy molecules resulted in a marginal increase in released stress for the initial first minutes (Figure 5B, C), going from ∼3% to 6% regarding **UPy**_*long*_ **-N**_**3**_ and **UPy**_*short*_ **-N**_**3**_, respectively. Interestingly, **UPy**_*long*_ **-N**_**3**_ showed a marginal increase in released stress (∼12%) at very long time scales (10,000 s), which is not observed in the other networks. This might indicate that in general, the star-based PEG network is dominant in the bulk dynamics, and **UPy**_*intermediate*_ **-N**_**3**_ and **UPy**_*short*_ **-N**_**3**_ do not alter the bulk stress relaxation as their fibers and clusters are not large enough to alter the bulk dynamics. Nevertheless, **UPy**_*long*_ **-N**_**3**_ is able to form very large clusters and fibers and thus might be able to show a small effect on the stress-relaxation behavior, especially at longer time-scales. This in accordance with previous results where UPy-based hydrogels show a very slow stress relaxation at longer time scales.^19,20,37^

**Figure 5:**
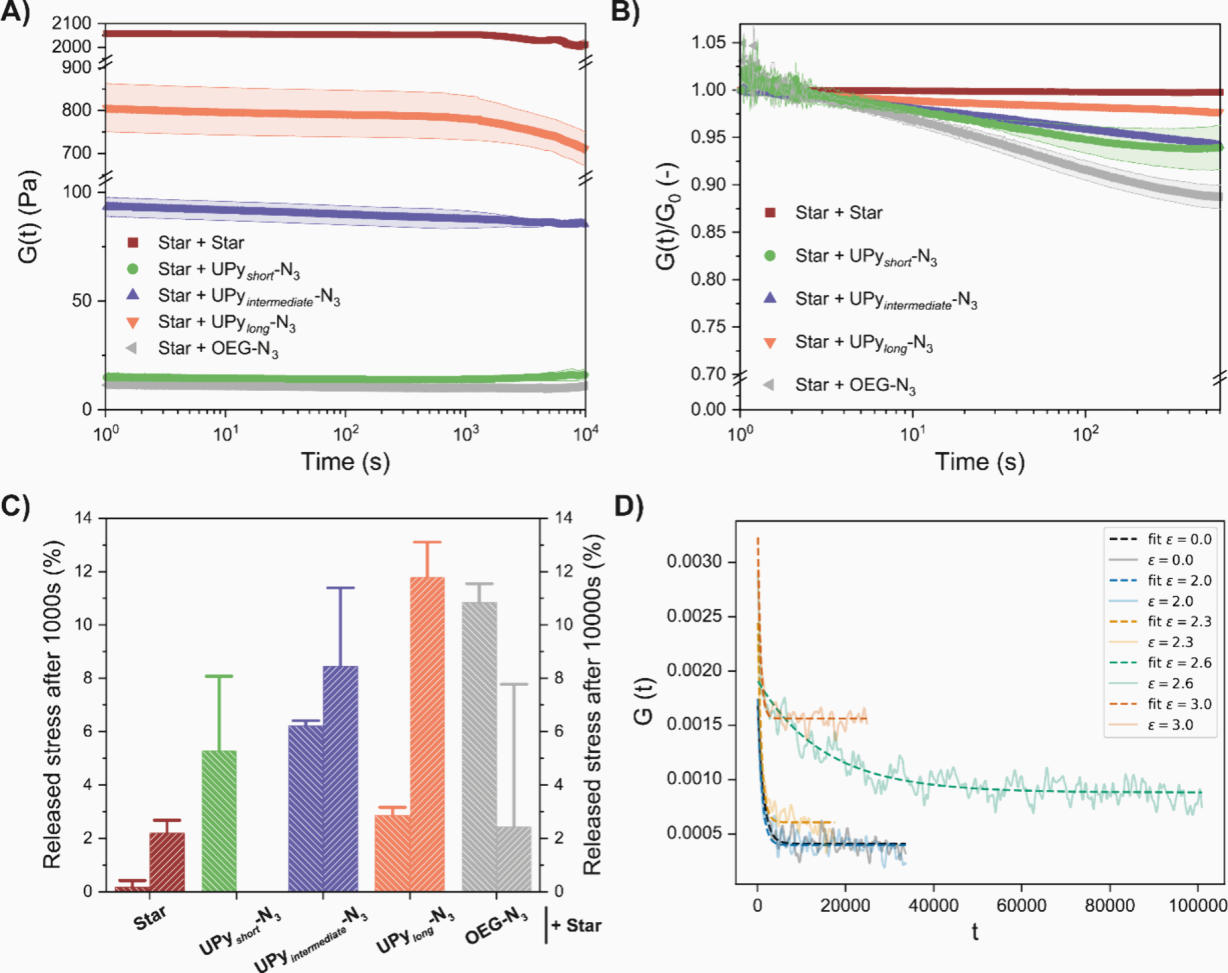
Dynamical properties of the various hydrogels. (A) Stress relaxation of the various networks, following the released stress over 10,000 s. Initially, a similar behavior is observed in terms of time-dependent stress decay, and only small differences are obtained at long time scales. (B) Normalized stress relaxation of the first few minutes. The results show that incorporation of longer UPy’s very marginally increases the dynamics of the network, i.e., faster stress relaxation. (C) Total released stress after 1000 and 10,000 s for the various networks. (D) Theoretical prediction of stress relaxation for the various networks. Simulations were performed using different interaction strengths between the UPy’s (ε = 0.0, 2.0, 2.3, 2.6, and 3.0).

For the more dynamic **UPy**_*short*_ **-N**_**3**_, *G*(*t*) increases even over very long time scales. This increase might indicate rearrangement and formation of new cross-links over time. Nevertheless, the differences are rather small and given the very low absolute values of *G*(*t*), any conclusions should be considered with care. In Figure 5D, the stress relaxation moduli obtained from the corresponding simulations are shown. All numerical stress relaxation curves show a rapid initial relaxation, which is too fast to capture in the experiments. Beyond the initial relaxation, there is clearly an effect of the interaction strength on the relaxation behavior. When a strong interaction is used, especially ε = 2.6, it takes longer to reach the plateau modulus, while for a weak interaction strength, the network behaves as a fluid and the ultimate relaxation goes toward zero.

Overall, this suggests that although the gels become softer when shorter UPy’s are incorporated, the dynamic behavior of the network in terms of stress relaxation is still mostly determined by the covalent bonds and the star-based PEG network. Interestingly, in experiments with **UPy**_*long*_ **-N**_**3**_ and simulations for ε = 2.6, both showed relaxation behavior at long time scales. In accordance, when looking at the longest time scales, we see that this time becomes significantly larger for ε = 2.6, indicating slowly relaxing clusters once the interaction strength is high enough (Figure S22). We hypothesize that once the interaction is strong enough or when long UPy’s are used, certain dynamic interactions are incorporated that can only be probed on long time scales. Furthermore, the simulations showed an interesting regime, once the interaction strength becomes very strong (ε = 3.0), the plateau modulus is reached very quickly. In this case, the simulation time is not long enough to capture the relaxation of the reversible bonds, which then behave essentially like irreversible bonds, and thus, the relaxation to a solid plateau is probed, which is much shorter.

Similar to what we did before, additional experiments were conducted in which various amounts of **Star-N**_**3**_ were replaced with **UPy**_*long*_ **-N**_**3**_ (Figure S23). Again, only a small increase in the normalized amount of released stress was observed during the stress relaxation experiments (Figure S23). These small differences further confirm that the bulk dynamic properties of the network are mostly dictated by the covalent bonds, even if their relative amount decreases.

## Conclusions

Overall, by using a fully synthetic hydrogel, we obtained tunable mechanics by using a combination of covalent and dynamic cross-links in the network. A purely covalent star network was used as the backbone, in which covalent cross-links were replaced by dynamic UPy cross-links. In this respect, by molecular changes in the UPy cross-links, the mechanics of network could be tuned over several orders of magnitude, all while maintaining the same concentration of the components. Coarse-grained molecular dynamics simulations showed a similar relationship between mechanics and interaction strength, as was obtained in the experiments between mechanics and UPy length. These results show the possibility to create networks with large different mechanical properties, all while keeping the global network components and concentrations similar, but by changing the molecular design and interaction strength of the cross-links. These design principles bring synthetic materials closer to tunability of the native ECM, in which both covalent and noncovalent interactions play a role.

## Supplementary Material



## Notes

Experimental section; synthetic route for the various UPy’s; rheology of the formation of the various hydrogels; degree of clustering with various amounts of UPy’s; prediction of moduli at various shear strains; rheological analysis with various amounts of **UPy_*long*_-N_3_**; representation of modular simulations for stress relaxation; simulations for varying amounts of UPy interaction strength and varying amounts of UPy (PDF)

## †

ASML 5504 DR Veldhoven, The Netherlands

## ‡

SGS, P.O. Box 2152, 1211 Geneva, Switzerland

## ABBREVIATIONS

UPy: ureido-pyrimidinone
ECM: extracellular matrix
PEG: poly­(ethylene glycol)
SPAAC: strain-promoted azide–alkyne click reaction
DCM: dichloromethane
Mw: polymer molecular weight
BCN: bicyclo­[6.1.0]­non-4-yne
BHT: butylated hydroxytoluene
HCl: hydrochloric acid
LAMMPS: Large-scale Atomic/Molecular Massively Parallel Simulator
WCA: Weeks–Chandler–Anderson potential
MD: molecular dynamics
LJ: Lennard-Jones
