# Supplementary material for: Controlling the Physical Properties in Hybrid Hydrogel Networks via Tunable Supramolecular Interactions
Source: Macromolecules. 2025 Jun 3;58(12):6077–87. doi: 10.1021/acs.macromol.5c00226 (PMC12203420; doi:10.1021/acs.macromol.5c00226)
Supplement: Supplementary file 1 [file ma5c00226_si_001.pdf]

Supporting information for

Controlling physical properties in hybrid hydrogel networks via tunable supramolecular interactions

Martin G.T.A. Rutten[1,2], *Chiara Raffaelli*[1,3,†], *Maxime O. Grillaud*[1,2,†], *Riccardo Bellan*[1,2], *Wouter G. Ellenbroek*[1,3], *Patricia Y.W. Dankers*[1,2,4 *]

[1] Institute for Complex Molecular Systems, Eindhoven University of Technology, P.O. Box 513, 5600 MB Eindhoven, The Netherlands

[2] Department of Biomedical Engineering, Laboratory of Chemical Biology, Eindhoven University of Technology, P.O. Box 513, 5600 MB Eindhoven, The Netherlands

[3] Department of Applied Physics, Eindhoven University of Technology, P.O. Box 513, 5600 MB Eindhoven, The Netherlands

[4] Department of Chemical Engineering and Chemistry, Eindhoven University of Technology, P.O. Box 513, 5600 MB Eindhoven, The Netherlands

Table of contents

1. **Instrumentation** ....Error! Bookmark not defined.
2. **Synthesis of UPy_{short}-N_3 and $UPy_{intermediate}$-N_3** 4
3. **Synthesis of UPy_{long}-N_3** 8
4. **Hydrogel composition**...Error! Bookmark not defined.
5. **Hydrogel formation** 18
6. **Cluster size and growth**.. 19
7. **Modulus at various shear strains** 20
8. **Changing the amount of UPy in the network**.. 21
9. **References**... 24

1. Instrumentation

All starting materials, chemicals, and solvents were obtained from commercial suppliers. All solvents were of AR quality and purchased from Biosolve. Deuterated compounds were obtained from Cambridge Isotope Laboratories and stored over 4 Å molecular sieves. Dry solvents were obtained using MBraun solvent purification system (MB SPS-800). Water for aqueous samples was purified on an EMD Millipore Milli-Q Integral Water Purification System. Dodecaethylene glycol and **OEG-N$_3$** were purchased from Polypure. **Star-N$_3$** was purchased from Jenkem (4arm-PEG$_{10k}$-N$_3$, MW ~ 10 kDa). **Star-BCN** was synthesized by SymoChem according to reported procedures[1] and stored while dissolved in DCM with 1 mg BHT as stabilizer to prevent crosslinking. The UPy-C6-NCO used for the synthesis of **UPy$_{long}$-N$_3$** was provided by Jolanda Spiering. Glassware was dried in an oven at 135 °C overnight prior to reactions under dry conditions. Reactions were followed by thin-layer chromatography (TLC) using 60-F254 silica gel plates from Merck and visualized by UV light at 254 nm and/or staining (ninhydrin, potassium permanganate). Flash column chromatography was performed on a Grace Reveleris X2 chromatography system using Reveleris Silica Flash cartridges. All ^{1}H-NMR spectra were recorded on Bruker Ultrashield spectrometers (400 MHz for ^{1}H NMR). Proton chemical shifts are reported in ppm (δ) downfield from trimethyl silane (TMS) using the resonance frequency of the deuterated solvent ($CDCl_3$; 7.26 ppm) as the internal standard. Peak multiplicities are abbreviated as s: singlet; dt; doublet of triplet; m: multiplet. Liquid chromatography mass spectroscopy (LC-MS) spectra were acquired using a device consisting of multiple components: Shimadzu SCL-10 A VP system controller with Shimadzu LC-10AD VP liquid chromatography pumps (with an Alltima C18 3 u (50 × 2.1 mm) reversed-phase column and gradients of water), a Shimadzu DGU 20A3 prominence degasser, a Thermo Finnigan surveyor auto sampler, a Thermo Finnigan surveyor PDA detector and a Thermo Scientific LCW Fleet. All samples were dissolved in 1:1 H_2O:ACN in *ca*. 0.1 mg mL^{-1} concentration. High-performance liquid chromatography-mass spectrometry (HPLC-MS) analyses were executed on a Shimidazu SCL-10 AD VP series HPLC coupled to a diode array detector (Finnigan Surveyor PDA Plus, Thermo Electron Corporation) and an Ion-Trap (LCQ Fleet Thermo Scientific). Infrared (IR) spectra were recorded on a Perkin–Elmer Spectrum One ATR-FTIR spectrometer.

2. Synthesis of UPy*short*-N_3 and UPy*intermediate*-N_3

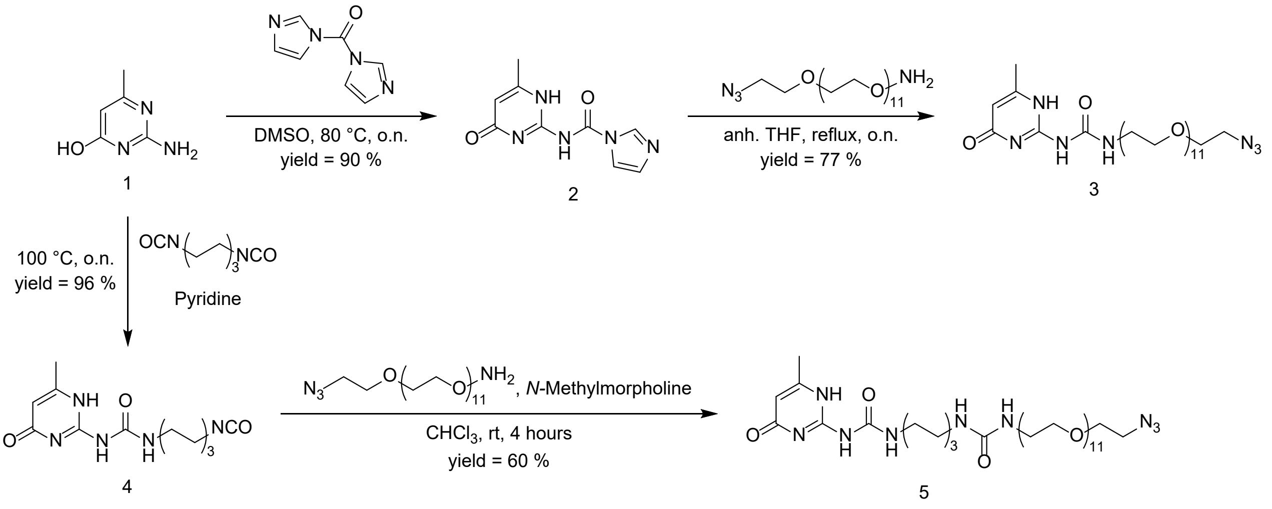

Scheme 1: Synthetic pathway towards **UPy*short*-N_3** and (compound 3) and **UPy*intermediate*-N_3** (compound 5).

N-(6-methyl-4-oxo-1,4-dihydropyrimidin-2-yl)-1H-imidazole-1-carboxamide (2)

A round bottom flask was charged with 2-amino-4-hydroxy-4-methylpyrimidine (168 mg; 1.34 mmol), 1, 1'-carbonyldiimidazole (296 mg; 1.83 mmol) and DMSO (2.0 mL). The resulting suspension was stirred overnight at 80 °C under Argon atmosphere. After 20 hours the mixture was cooled down to room temperature and the formed solid was suspended in acetone (5.0 mL), filtered and washed with acetone (3 × 5.0 mL). The final compound was dried under vacuum and 264 mg were received as a white powder in 90 % yield. As a consequence of the poor solubility of the received compound in most of the available solvents, it was used directly used in the next step without any characterization.

1-(32-azido-3,6,9,12,15,18,21,24,27,30-decaoxadotriacontyl)-3-(6-methyl-4-oxo-1,4-dihydropyrimidin-2-yl)urea (3)

Compound 2 (64 mg; 0.29 mmol) was suspended in anhydrous THF (0.5 mL) and a solution of azido-PEG_{11}-amine (119 mg; 0.21 mmol) in anhydrous THF (0.3 mL) was added dropwise while stirring under Argon atmosphere. Additional solvent was added (0.7 mL) and the mixture was heated to reflux overnight while stirring. After 20 hours the mixture was cooled down to room temperature and the excess of 2 was quenched with a drop of aqueous hydrogen chloride (HCl) (2 M). After the solvent was removed under vacuum, water was added (5.0 mL) and the resulting mixture was acidified with aqueous HCl 2 M until pH around 5.0. The aqueous layer was extracted with DCM (3 × 10 mL) and the combined organic layers were dried over $MgSO_4$, filtered and concentrated under vacuum. The crude product was purified through normal-phase flash chromatography (Eluent: DCM/MeOH = 95/5) to afford 151 mg of the desired compound as a wax in 77 % yield. ^{1}H-NMR (400 MHz, Chloroform-*d*) δ 13.03 (s, 1H), 11.90 (s, 1H), 10.25 (s, 1H), 5.80 (t, J = 1.4 Hz, 1H), 3.70 – 3.59 (m, 44H), 3.46 (dt, J_{HCNH} = 8.0 Hz, J_{HCNH} = 4.0 Hz 2H), 3.39 (t, J = 4.0 Hz, 2H), 2.23 (s, 3H). LC-MS: calc.: *m/z* 721.39, found: $[M+H]^+$ = 722.33.

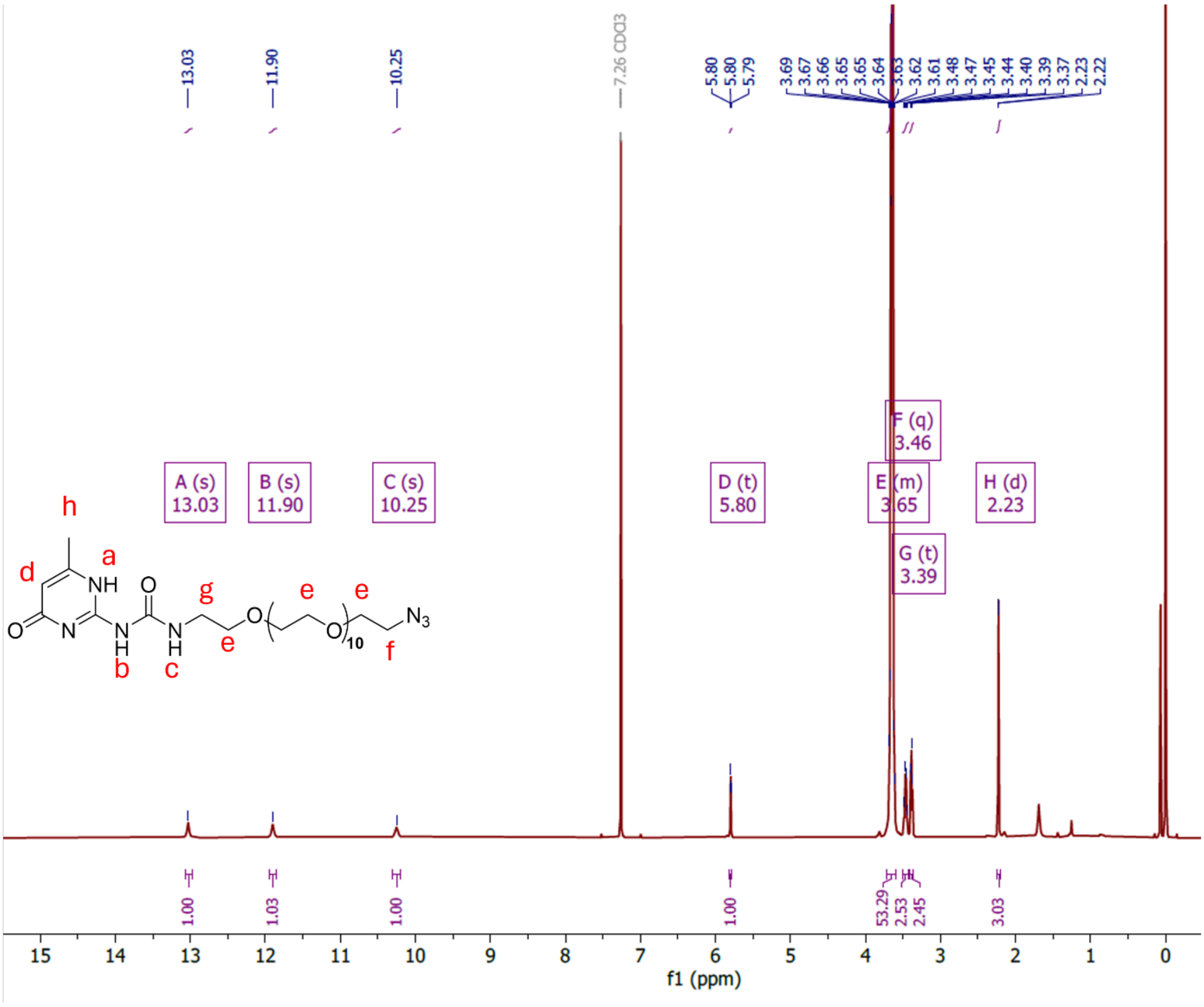

Fig. S1. ^{1}H-NMR (400 MHz) of compound **3** in $CDCl_3$.

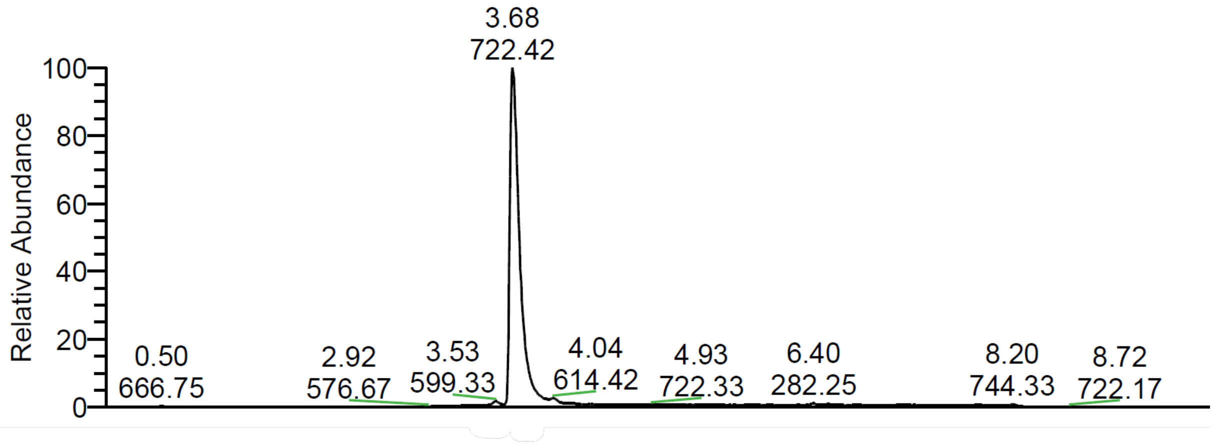

Fig. S2. LC- MS (positive) of compound **3**. *m/z* calculated: 721.39, found: $[M+H]^+$ = 722.42.

1-(2-isocyanatoethyl)-3-(6-methyl-4-oxo-1,4-dihydropyrimidin-2-yl)urea (4)

A round bottom flask was charged with 2-amino-4-hydroxy-4-methylpyrimidine (5.0 g; 40 mmol), hexamethylene diisocyanate (100 mL; 680 mmol) and pyridine (1.0 mL; 12 mmol). The resulting mixture was heated to 100 °C overnight under Argon atmosphere while stirring. After 16 hours the mixture was cooled to room temperature, diluted with 57.0 mL of heptane and filtered over buchner filter. During the filtration the buchner filter was covered with a large funnel with a N_2 flow to protect the final product from humidity. The product was washed with heptane (5 × 43 mL) and hexane (2 x 43 ml). The product was finally dried under vacuum to provide 11.3 g of the desired compound as a white powder in 96% yield. ^{1}H-NMR(400 MHz, $CDCl_3$): δ 13.11 (s, 1H), 11.86 (s, 1H), 10.19 (s, 1H), 5.82 (s, 1H), 3.30-3.23 (m, 4H), 2.23 (s, 3H), 1.65-1.58 (m, 6H), 1.48 – 1.33 (m, 2H). ^{13}C-NMR (100 MHz, $CDCl_3$): δ 173.22, 156.75, 154.86, 148.43, 106.85, 43.03, 39.93, 31.34, 29.45, 26.38, 26.32, 19.09.

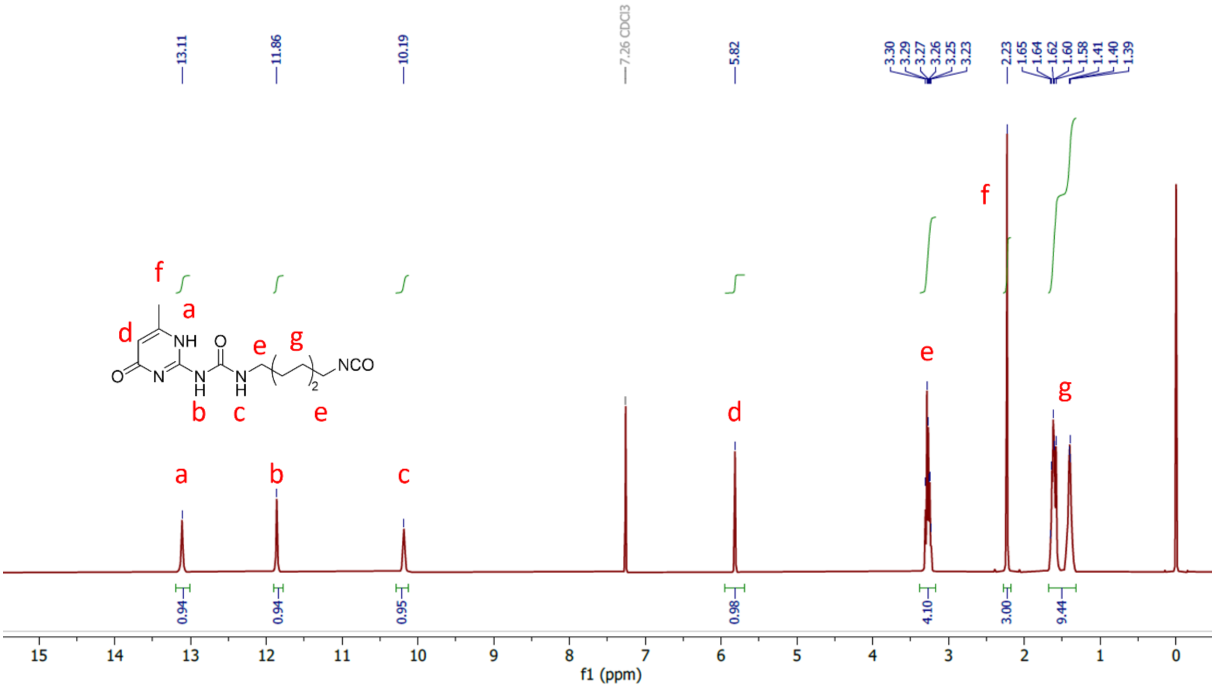

Fig. S3. ^{1}H-NMR (400 MHz) of compound **4** in $CDCl_3$.

1-(1-azido-34-oxo-3,6,9,12,15,18,21,24,27,30-decaoxa-33,35-diazahentetracontan-41-yl)-3-(6-methyl-4-oxo-1,4-dihydropyrimidin-2-yl)urea (5)

Compound 4 (88 mg; 0.3 mmol) was suspended in $CHCl_3$ (4.0 mL) along with *N*-methylmorpholine (60 µL; 0.55 mmol) and a solution of azido-PEG$_{11}$-amine (180 mg; 0.32 mmol) in $CHCl_3$ (2.0 mL) was added dropwise under Argon atmosphere while stirring. The mixture was allowed to stir at room temperature for 5 hours. Product formation was confirmed by the disappearance of the IR isocyanate band at 2270 cm^{-1}. The solvent was removed under vacuum and the crude product was dissolved in $CHCl_3$ (3.0 mL) and precipitated in Et_2O (45 mL). The precipitate was collected through centrifugation (4250 rpm, 10 min). The pellet was finally purified through normal-phase flash-chromatography (Eluent:

DCM/MeOH/ethylene glycol dimethyl ether = 8/1/1) to afford 148 mg of the desired compound as a white powder in 60 % yield. ^{1}H-NMR (400 MHz, Chloroform-*d*) δ 13.13 (s, 1H), 11.85 (s, 1H), 10.12 (s, 1H), 5.83 (s, 1H), 5.19 (s, 1H), 5.05 (s, 1H), 3.64 (td, *J* = 9.3, 8.2, 5.1 Hz, 41H), 3.38 (dt, *J* = 9.2, 5.0 Hz, 4H), 3.24 (d, *J* = 6.5 Hz, 2H), 3.14 (d, *J* = 6.6 Hz, 2H), 2.23 (s, 3H), 1.48 (s, 2H), 1.36 (s, 3H). ^{1}H NMR (400 MHz, Chloroform-*d*) δ 13.13 (s, 1H), 11.85 (s, 1H), 10.12 (s, 1H), 5.83 (s, 1H), 5.19 (s, 1H), 5.05 (s, 1H), 3.68 – 3.61 (m, 42H), 3.55 (t, *J* = 4.0 Hz, 2H), 3.40 – 3.35 (mj, 4H), 3.24 (dt, *J* = 8.0 Hz, *J* = 4.0 Hz, 2H), 3.14 (dt, *J* = 8.0 Hz, *J* = 4.0 Hz, 2H), 2.23 (s, 3H), 1.50 - 1.36 (m, 8H). LC-MS: calc.: *m/z:* 863.50.51; found: $[M+H]^+$ = 864.42.

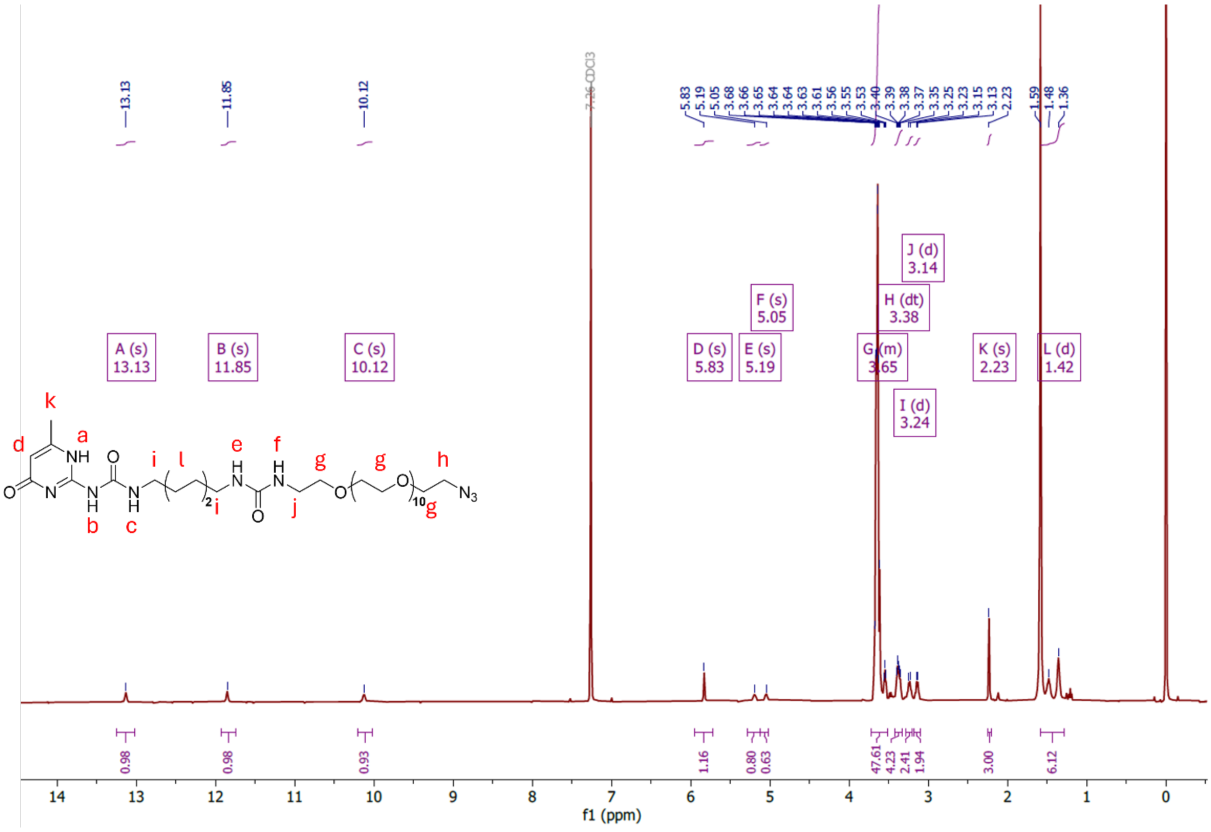

Fig. S4. ^{1}H-NMR (400 MHz) of compound **5** in $CDCl_3$.

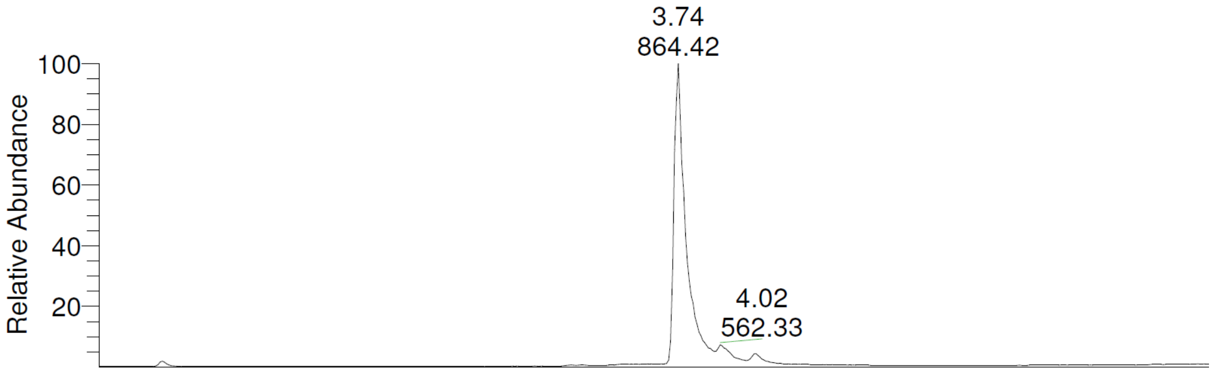

Fig. S5. LC- MS (positive) of compound **5**. *m/z* calculated: 852.51, found: $[M+H]^+$ = 863.50, $[M+H]^+$ = 864.42.

3. Synthesis of UPy$_{long}$-N$_3$

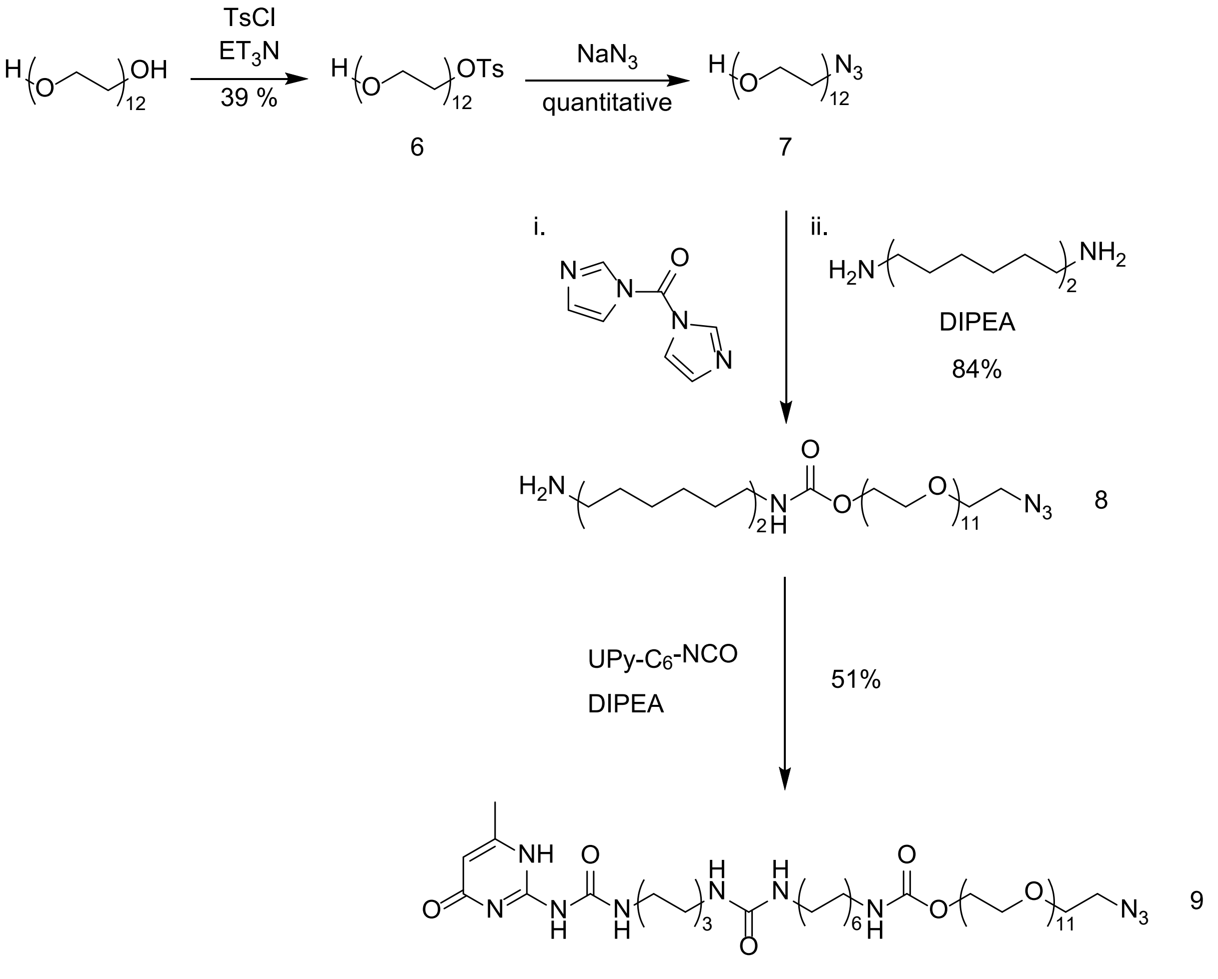

Scheme 2. Synthetic pathway towards **UPy$_{long}$-N$_3$** (compound 9).

HO-PEG$_{12}$-OTs (6)

Dodecaethylene glycol (2 g, 3.66 mmol, 1x) was dissolved in DCM 30 mL and triethylamine (Et_3N, 765 µL, 5.49 mmol, 1.5x) was added to the solution. 4-Toluenesulfonyl chloride (TsCl, 907 mg, 4.76 mmol, 1.3x) was dissolved in DCM 30 mL and added dropwise to the stirred mixture. The reaction was stirred overnight at room temperature. 300 mL of DCM were added to the reaction mixture and the organic phase was washed with brine (2x400 mL), dried over Na_2SO_4 and filtered. The solvent was removed under reduced pressure and the crude product was purified by column chromatography on silica gel (eluent: AcOEt/MeOH 100:00 to 95:05) affording **6** as a pale yellow oil (998 mg, 39 % yield). ^{1}H NMR ($CDCl_3$) δ: 7.80 (d, 2x 1H, ^3J = 8 Hz, 2x Ar*H*), 7.35 (d, 2x 1H, ^3J = 8 Hz, 2x Ar*H*), 4.16 (t, ^3J = 4.8 Hz, 2H, -CH_2-OTs), 3.75-3.57 (m, 46H, 23x -CH_2O-), 2.70 (br s, 1H, -O*H*), 2.45 (s, 3H, Ar-CH_3); ^{13}C NMR ($CDCl_3$) δ: 144.94, 133.13, 129.98, 128.13, 72.67, 70.89, 70.70, 70.46, 69.41, 68.82, 61.86, 21.81. FT-IR (ATR) ν (cm^{-1}): 3485, 2869, 1644, 1598, 1454, 1351, 1293, 1248, 1189, 1176, 1094, 1038, 1019. HPLC-MS: 5.44 min, *m/z*: 701.25 $[M+H]^+$, 723.33 $[M+Na]^+$.

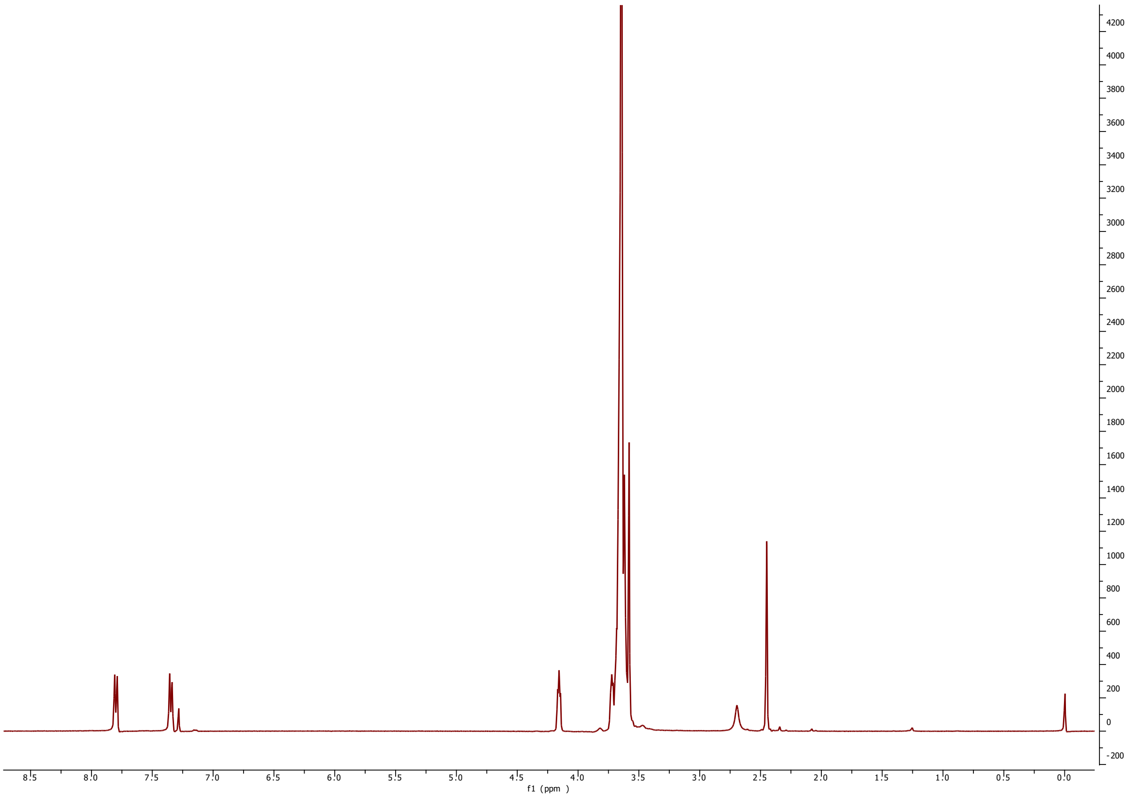

Figure S6. ^{1}H NMR spectrum ($CDCl_3$, 400 MHz) of **6**.

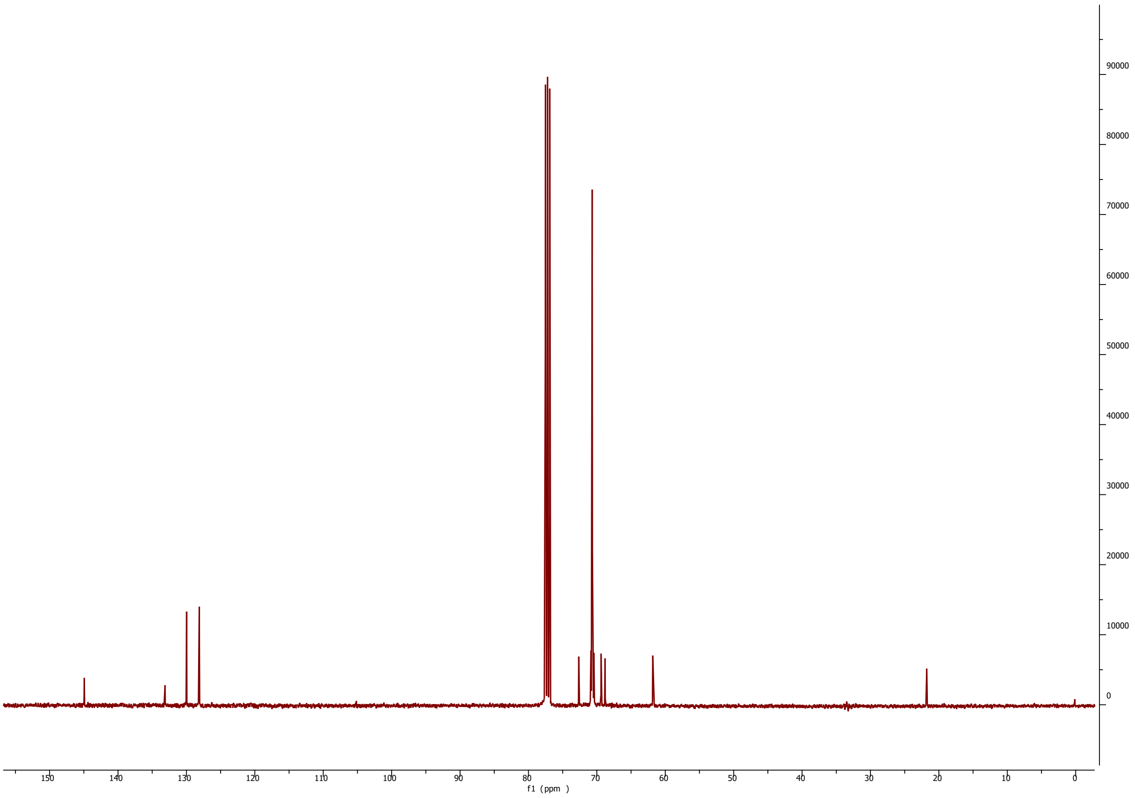

Figure S7. ^{13}C NMR spectrum ($CDCl_3$, 400 MHz) of **6**.

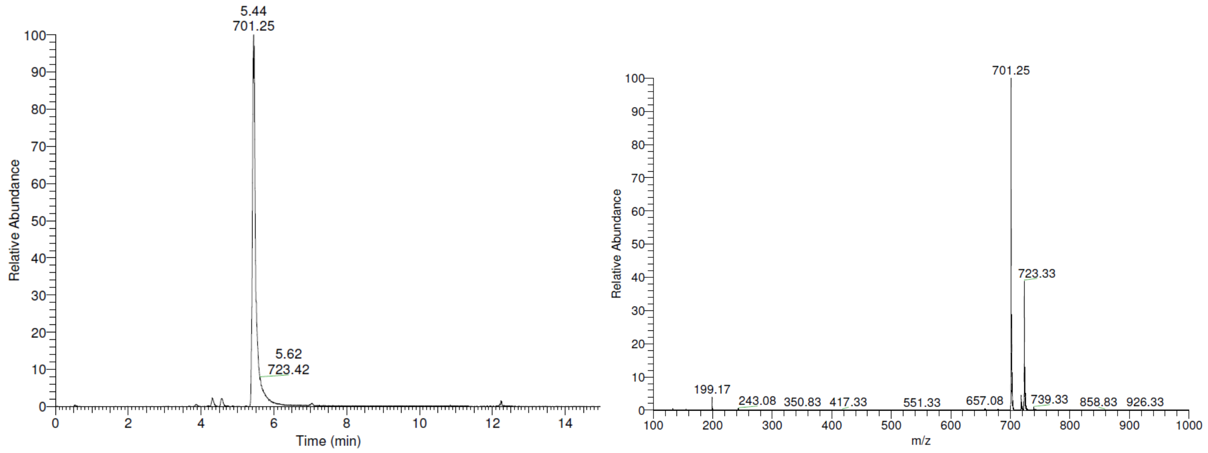

Figure S8. HPLC chromatogram - mass spectrum of **6**.

HO-PEG$_{12}$-N$_3$ (7)

Compound **6** (950 mg, 1.36 mmol, 1x) in a dried flask was dissolved in dry DMF 30 mL. Sodium azide (NaN_3, 176 mg, 2.72 mmol, 2x) was dissolved in dry DMF 20 mL and added to the solution. The reaction was stirred overnight at 70 °C under an argon atmosphere and 50 mL of H_2O was slowly added to the reaction mixture. After extractions with DCM (3x100 mL), the organic phases were washed with brine (2x200 mL), dried over Na_2SO_4 and filtered. The solvent was removed under reduced pressure giving **7** as a clear oil (756 mg, quantitative yield). ^{1}H NMR ($CDCl_3$) δ: 3.75-3.59 (m, 46H, 23x -CH_2O-), 3.39 (t, ^3J = 5.2 Hz, 2H, -CH_2-N_3), 2.68 (br s, 1H, -O*H*); ^{13}C NMR ($CDCl_3$) δ: 72.68, 70.86, 70.83, 70.80, 70.78, 70.74, 70.52, 70.20, 61.90, 50.84. FT-IR (ATR) ν (cm^{-1}): 3493, 2867, 2102, 1643, 1454, 1348, 1296, 1249, 1095. HPLC-MS: 4.66 min, *m/z*: 572.17 $[M+H]^+$, 594.33 $[M+Na]^+$.

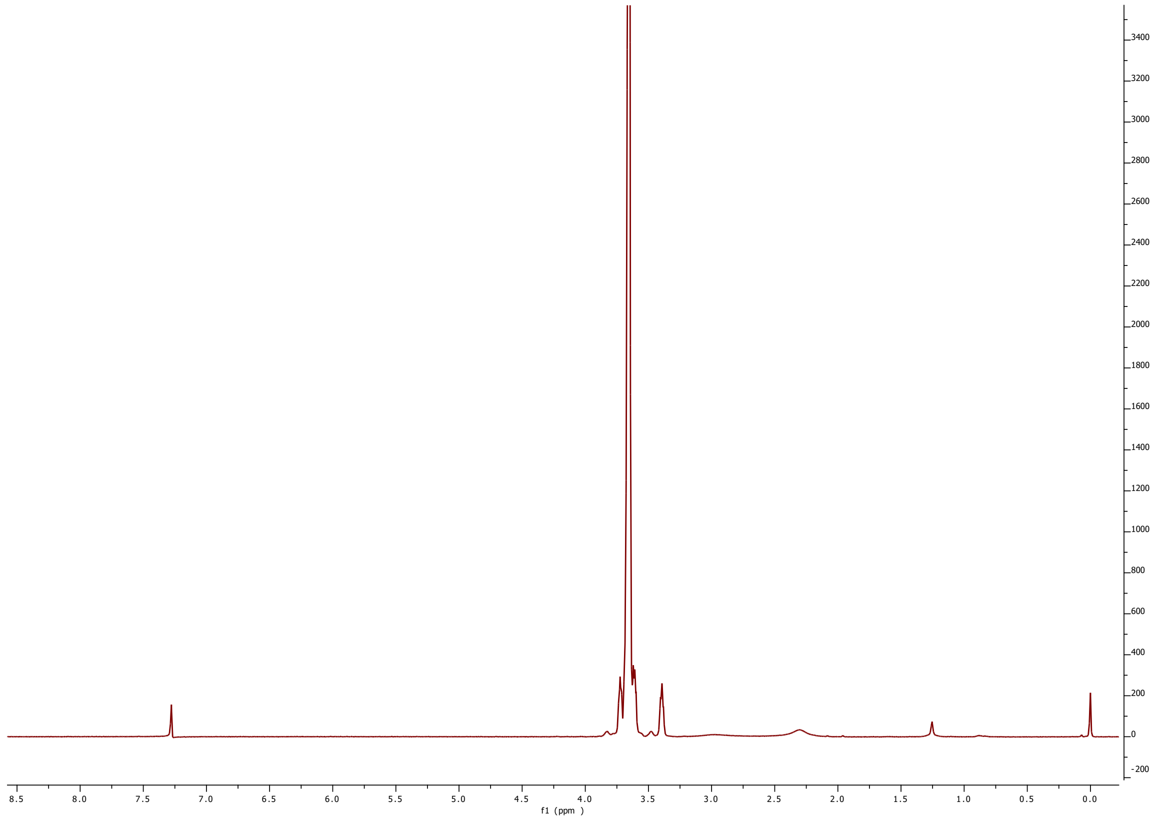

Figure S9. ^{1}H NMR spectrum ($CDCl_3$, 400 MHz) of **7**.

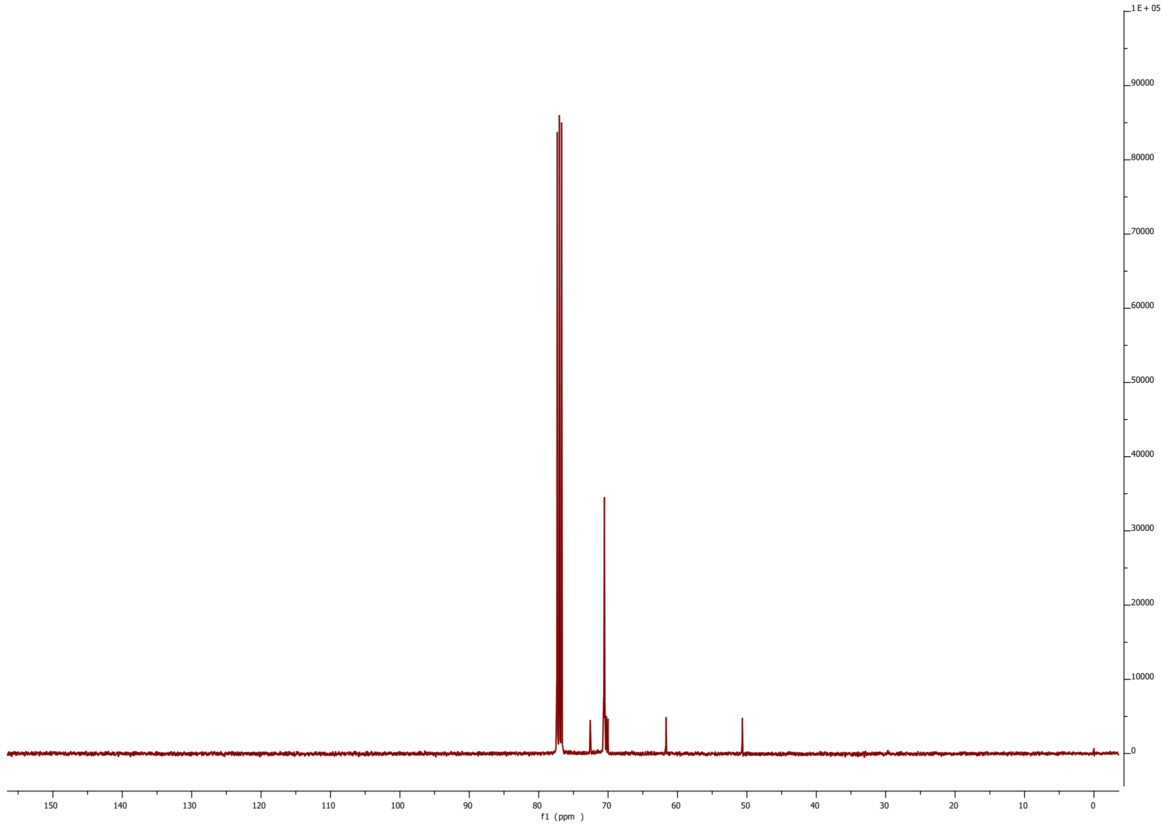

Figure S10. ^{13}C NMR spectrum ($CDCl_3$, 400 MHz) of **7**.

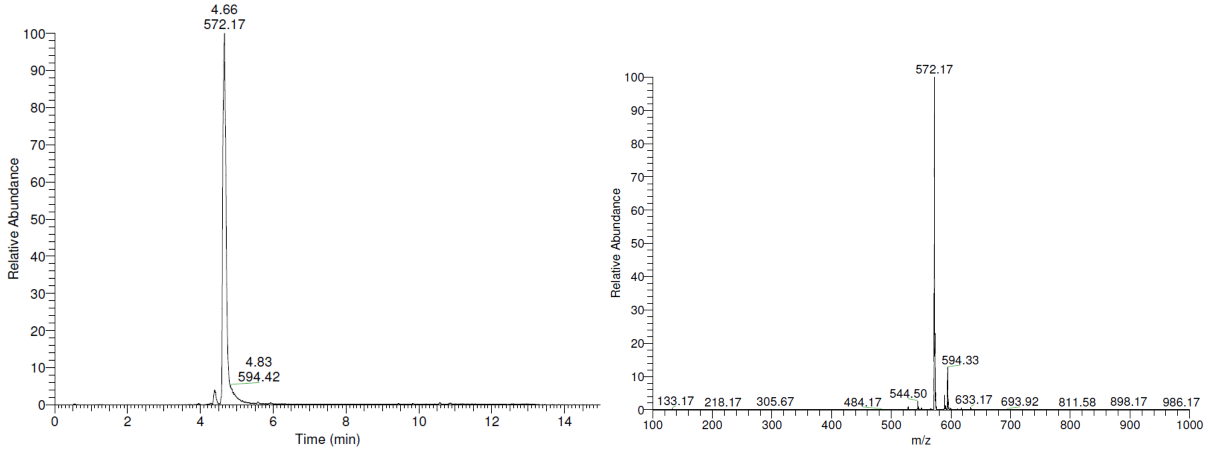

Figure S11. HPLC chromatogram - mass spectrum of 7.

H_2N-U-C_{12}-PEG_{11}-N_3 (8)

Compound **7** (500 mg, 0.88 mmol, 1x) in a dried flask was dissolved in $CHCl_3$ 4 mL. 1,1'-Carbonyldiimidazole (CDI, 284 mg, 1.76 mmol, 2x) was dissolved in $CHCl_3$ 3 mL and added to the mixture. The reaction was stirred 2 hrs at room temperature under an argon atmosphere, the complete synthesis of the CDI-activated intermediate was followed by HPLC-MS. 1,12-Diaminododecane (1.402 g, 7.00 mmol, 8x) in another dried flask was dissolved in $CHCl_3$ 20 mL and *N*,*N*-diisopropylethylamine (DIPEA, 3.05 mL, 17.49 mmol, 20x) was added to the diamine solution. The CDI-activation mixture was added to the diamine mixture and the reaction was stirred overnight at 80 °C under an argon atmosphere. $CHCl_3$ was removed under reduced pressure and the crude product was directly purified by column chromatography on silica gel (eluent: DCM/MeOH 100:00 to 95:05) yielding **8** as a white solid (589 mg, 84 % yield). ^{1}H NMR ($CDCl_3$) δ: 7.03 (br s, 2H, -NH_2), 5.39 (t, ^3J = 5.6 Hz, 1H, -N*H*-), 4.14 (t, ^3J = 4 Hz, 2H, -NH-C(=O)OC$H_2$$CH_2$-), 3.70-3.48 (m, 44H, 22x -CH_2O-), 3.33 (t, ^3J = 5.2 Hz, 2H, -CH_2-N_3), 3.13-3.03 (m, 2H, -CH_2CH_2-NH-C(=O)O-), 2.84 (t, ^3J = 7.6 Hz, 2H, -CH_2-NH_2), 1.71-1.57 (m, 2H, -C$H_2$$CH_2$-NH-C(=O)O-), 1.48-1.37 (m, 2H, -C$H_2$$CH_2$-$NH_2$), 1.34-1.14 (m, 16H, 8x -CH_2C$H_2$$CH_2$-); ^{13}C NMR ($CDCl_3$) δ: 156.60, 70.73, 70.69, 70.66, 70.57, 70.56, 70.49, 70.09, 69.92, 63.68, 50.73, 41.11, 40.40, 29.95, 29.47, 29.44, 29.41, 29.25, 29.13, 28.47, 26.79, 26.72. FT-IR (ATR) ν (cm^{-1}): 3295, 2914, 2886, 2849, 2142, 2101, 1690, 1549, 1523, 1466, 1359, 1343, 1279, 1241, 1147, 1106, 1061. HPLC-MS: 5.51 min, *m/z*: 399.83 $[M+2H]^{2+}$, 798.67 $[M+H]^+$, 820.58 $[M+Na]^+$.

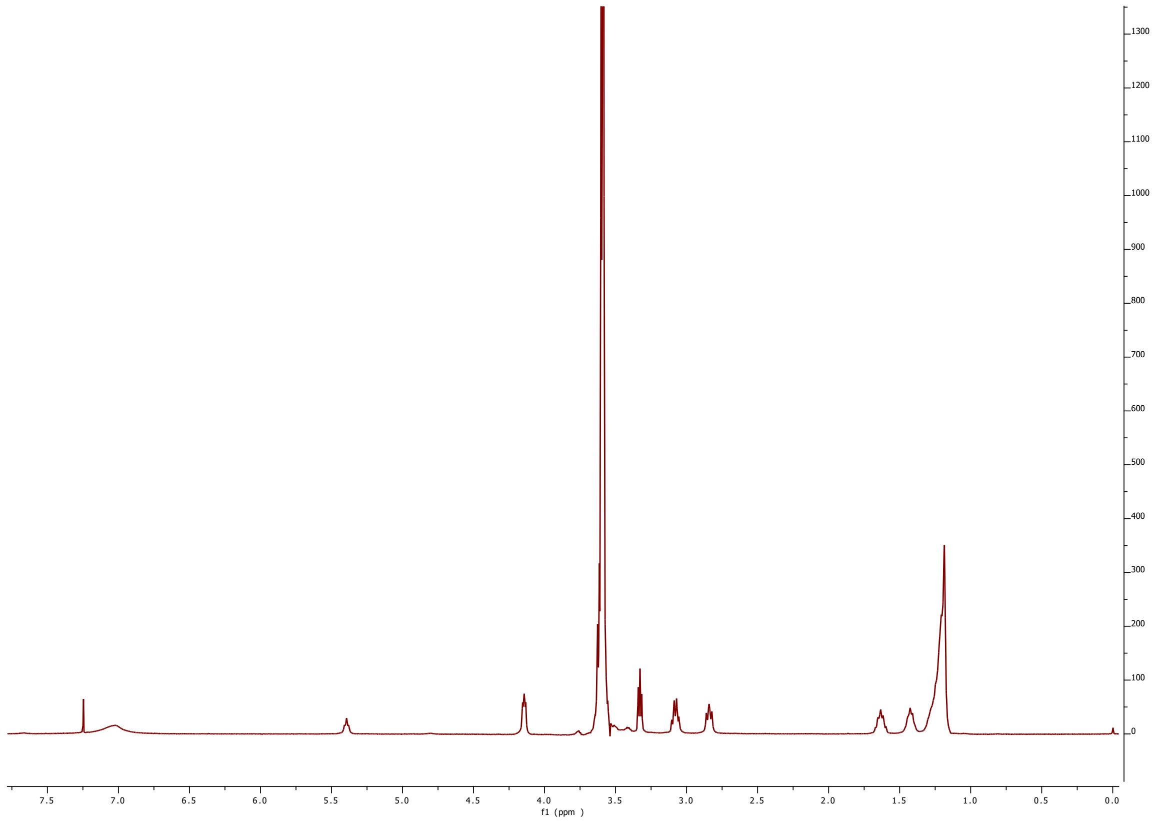

Figure S12. ^{1}H NMR spectrum ($CDCl_3$, 400 MHz) of **8**.

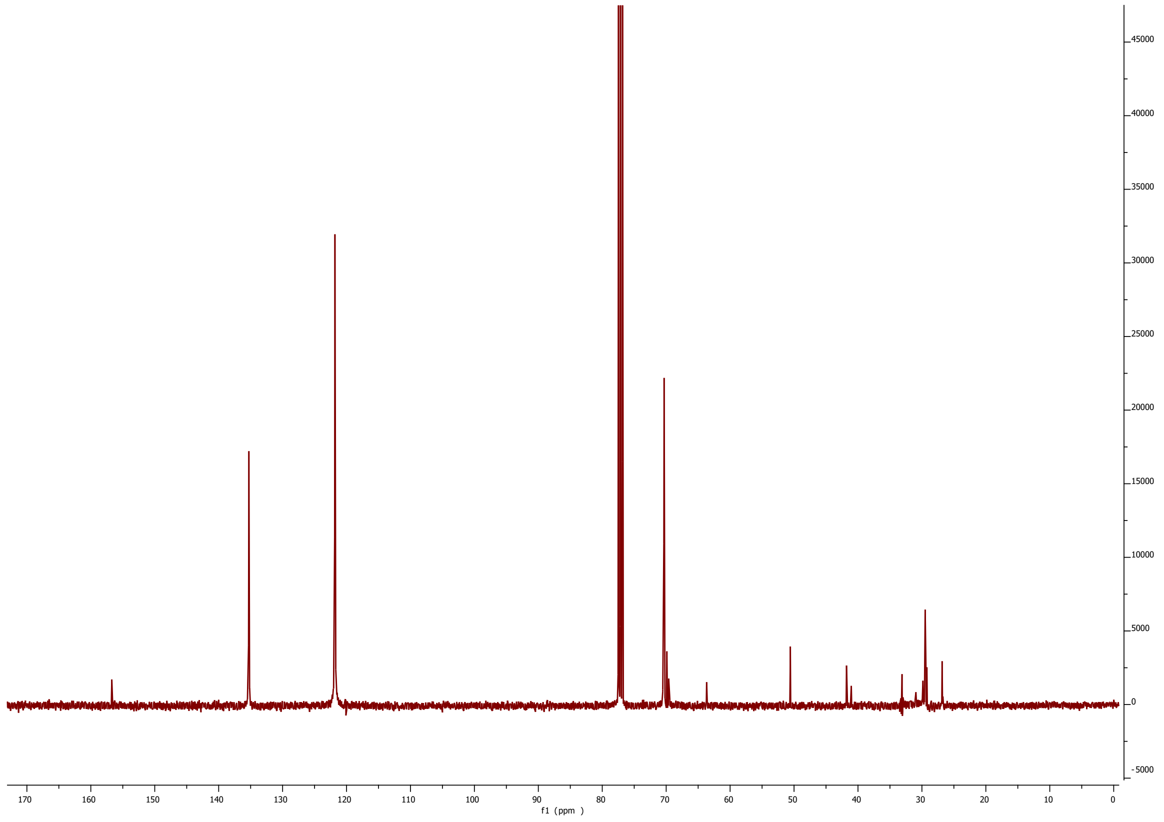

Figure S13. ^{13}C NMR spectrum ($CDCl_3$, 400 MHz) of **8**.

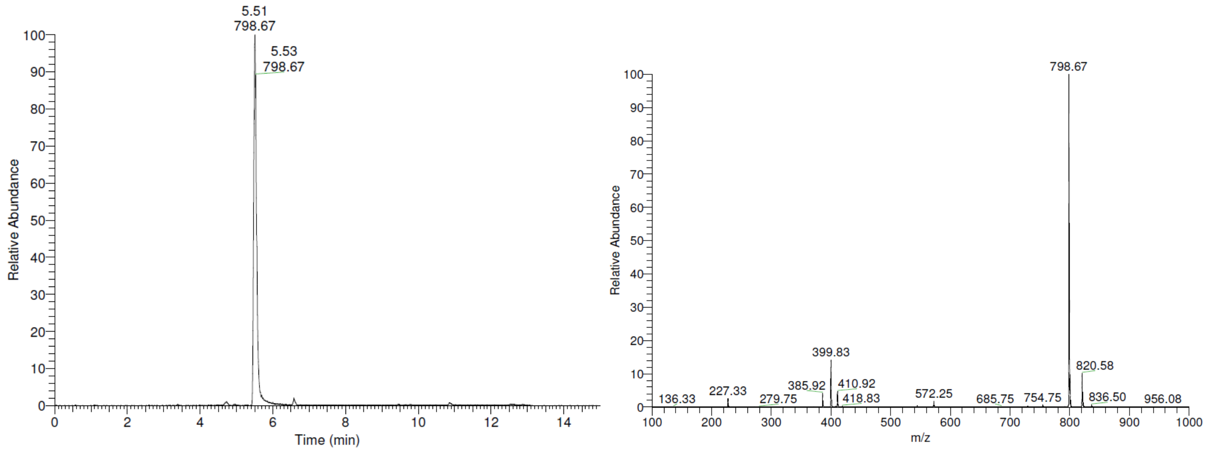

Figure S14. HPLC chromatogram - mass spectrum of **8**.

UPy-C_6-U-C_{12}-PEG_{11}-N_3 (9)

Compound **8** (100 mg, 0.125 mmol, 1x) and UPy-C6-NCO (40 mg, 0.138 mmol, 1.1x) in a dried flask were dissolved in $CHCl_3$ 25 mL. DIPEA (44 µL, 0.251 mmol, 2x) was added to the mixture and the reaction was stirred overnight at 50 °C under an argon atmosphere. $CHCl_3$ was removed under reduced pressure and the crude product was directly purified by reversed-phase column chromatography on C18 silica gel using a 30 min linear gradient (5-100 % of ACN and constant 1 % Et_3N). Lyophilization of pure fractions afforded **9** as a white solid (70 mg, 51 % yield). ^{1}H NMR ($CDCl_3$) δ: 13.17 (br s, 1H, N*H* UPy), 11.81 (br s, 1H, N*H* UPy), 10.08 (br s, 1H, N*H* UPy), 5.82 (s, 1H, =C*H*- UPy), 4.98 (t, ^3J = 5.2 Hz, 1H, N*H*), 4.93 (t, ^3J = 5.2 Hz, 1H, N*H*), 4.81 (t, ^3J = 5.2 Hz, 1H, N*H*), 4.20 (t, ^3J = 4.4 Hz, 2H, -NH-C(=O)OCH_2CH_2-), 3.75-3.59 (m, 44H, 22x -CH_2O-), 3.39 (t, ^3J = 5.2 Hz, 2H, -CH_2-N_3), 3.27-3.20 (m, 2H, -CH_2CH_2-NH-C(=O)O-), 3.19-3.11 (m, 6H, 3x -CH_2CH_2-NH-C(=O)NH-), 2.24 (s, 3H, -CH_3 UPy), 1.65-1.18 (m, 28H, 14x -CH_2CH_2CH_2-); ^{13}C NMR ($CDCl_3$) δ: 173.37, 158.81, 156.63, 154.88, 148.66, 106.68, 70.85, 70.82, 70.78, 70.72, 70.66, 70.19, 69.84, 63.94, 50.83, 41.20, 40.59, 40.19, 39.76, 29.99, 29.66, 29.40, 26.89, 26.35, 19.12. FT-IR (ATR) ν (cm^{-1}): 3539, 3330, 3213, 3140, 3035, 2924, 2853, 2103, 1701, 1667, 1620, 1582, 1530, 1463, 1442, 1415, 1349, 1303, 1256, 1100, 1043. HPLC-MS: 7.02 min, *m/z*: 546.42 $[M+2H]^{2+}$, 557.42 $[M+H+Na]^{2+}$, 568.42 $[M+2Na]^{2+}$.

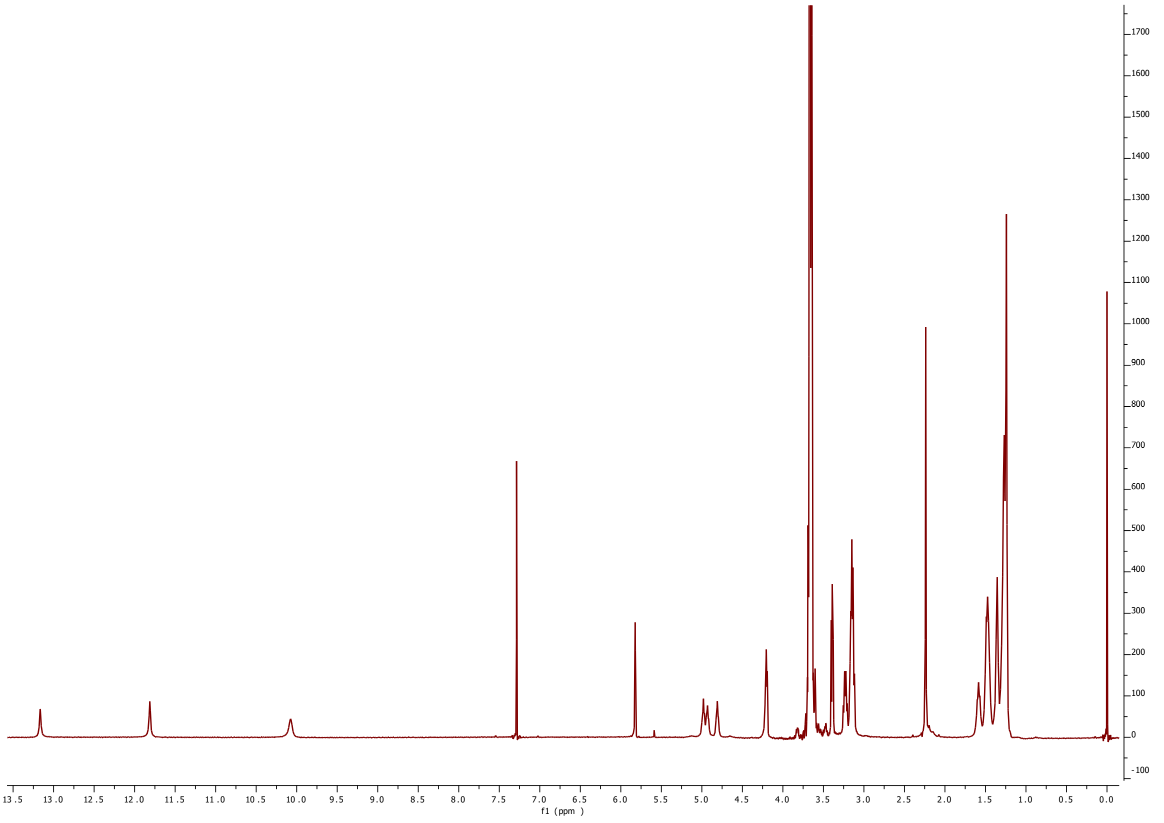

Figure S15. ^{1}H NMR spectrum ($CDCl_3$, 400 MHz) of **9**.

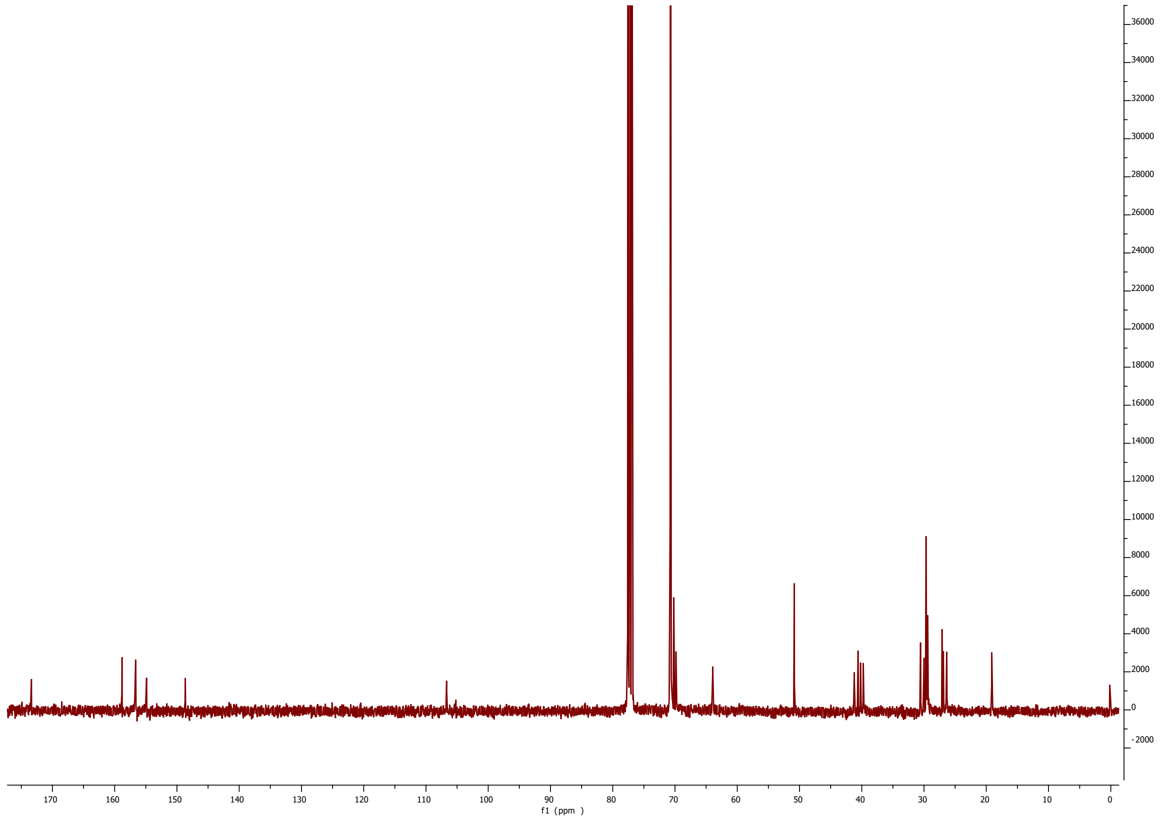

Figure S16. ^{13}C NMR spectrum ($CDCl_3$, 400 MHz) of **9**.

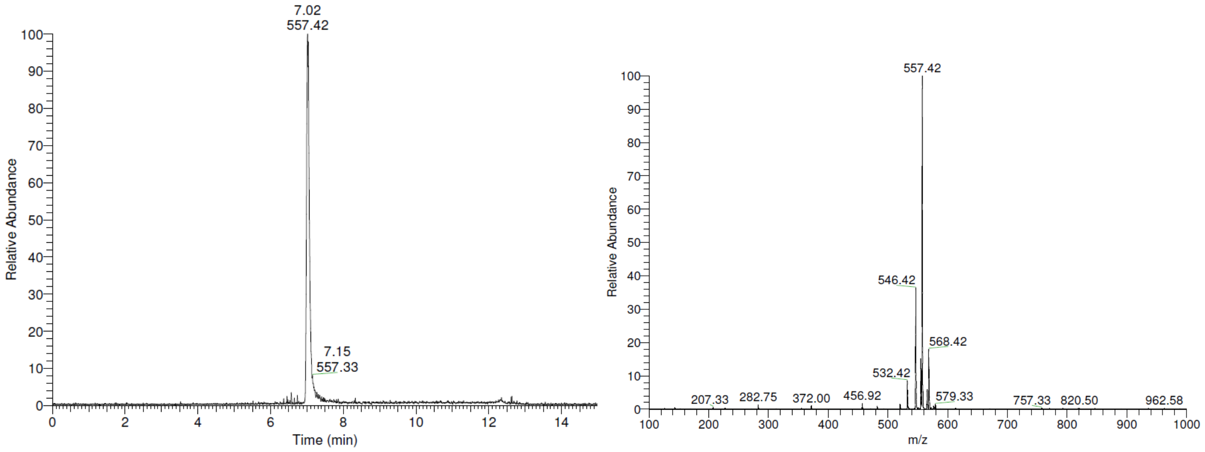

Figure S17. HPLC chromatogram - mass spectrum of **9**.

4. Hydrogel composition

Table S1. Table showing the composition of the hydrogels, containing different UPy's. In all cases the total concentration of the hydrogel was 3 wt%.

Sample ID	Concentration [mg mL^{-1}]	Molar concentration [mM]	Molar ratio [-]
	**(Star-BCN) – (Star-N_3) – (UPy-N_3 or OEG-N_3)**	**(Star-BCN) – (Star-N_3) – (UPy-N_3 or OEG-N_3)**	**(Star-BCN) – (Star-N_3) – (UPy-N_3 or OEG-N_3)**
**Star + Star**	15.7 – 14.3 – 0	1.39 – 1.39 – 0	1 – 1 – 0
**Star + UPy$_{short}$-N_3**	19.0 – 8.61 – 2.42	1.67 – 0.837 – 3.35	2 – 1 – 4
**Star + UPy$_{intermediate}$-N_3**	18.7 – 8.47 – 2.85	1.65 – 0.823 – 3.29	2 – 1 – 4
**Star + UPy$_{long}$-N_3**	18.2 – 8.27 – 3.51	1.61 – 0.803 – 3.21	2 – 1 – 4
**Star + OEG-N_3**	19.3 – 8.75 – 1.94	1.70 – 0.851 – 3.40	2 – 1 – 4

Table S2. Table showing the composition of the hydrogels, containing a different amount of UPy$_{long}$-N_3. In all cases the total concentration of the hydrogel was 3 wt%.

Sample ID	Concentration [mg mL^{-1}]	Molar concentration [mM]	Molar ratio [-]
	**(Star-BCN) – (Star-N_3) – (UPy$_{long}$-N_3)**	**(Star-BCN) – (Star-N_3) – (UPy$_{long}$-N_3)**	**(Star-BCN) – (Star-N_3) – (UPy$_{long}$-N_3)**
**Star + Star**	15.7 – 14.3 – 0	1.39 – 1.39 – 0	1 – 1 – 0
**Star + UPy$_{long}$-N_3 (A) (20% UPy$_{long}$-N_3)**	16.6 – 12.1 – 1.28	1.47 – 1.17 – 1.17	1 – 0.8 – 0.8
**Star + UPy$_{long}$-N_3 (B) (40% UPy$_{long}$-N_3)**	17.7 – 9.62 – 2.72	1.56 – 0.934 – 2.49	1 – 0.6 – 1.6
**Star + UPy$_{long}$-N_3 (C) (60% UPy$_{long}$-N_3)**	18.8 – 6.84 – 4.35	1.66 – 0.664 – 3.98	1 – 0.4 – 2.4

5. Hydrogel formation

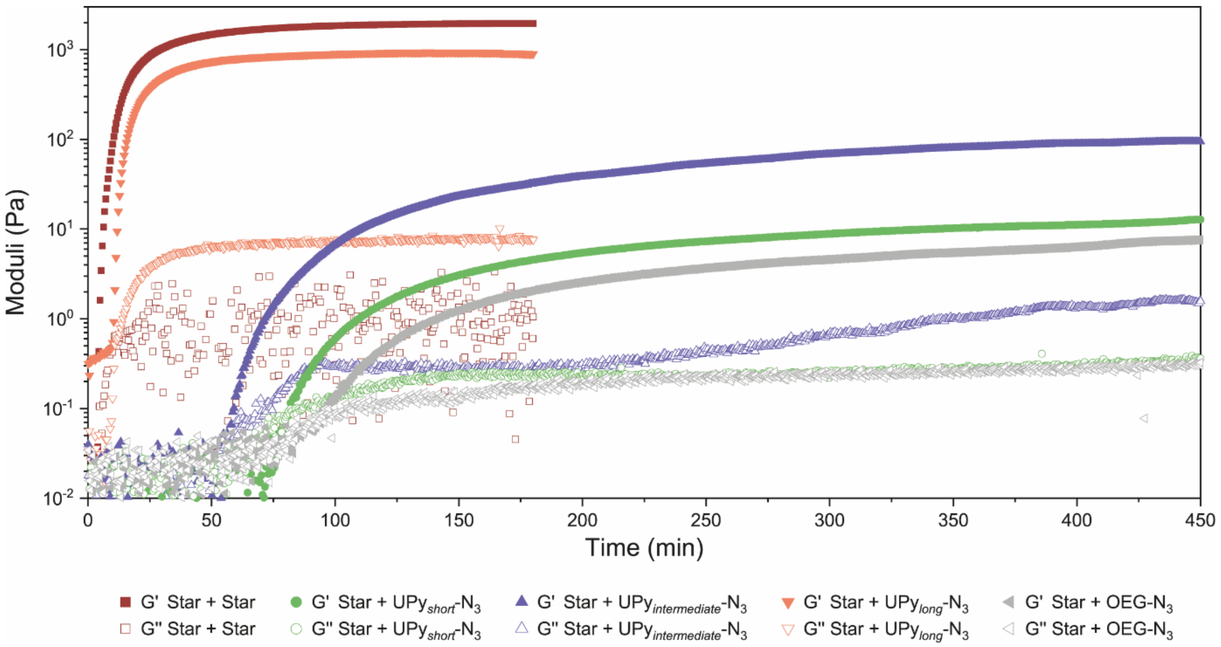

Figure S18. Formation time of the various hydrogel networks. The pure covalent network as well as the network with UPy$_{long}$-N$_3$ forms relatively fast. Incorporation of shorter UPy's increases the needed formation time and the networks remain liquid-like much longer.

6. Cluster size and growth

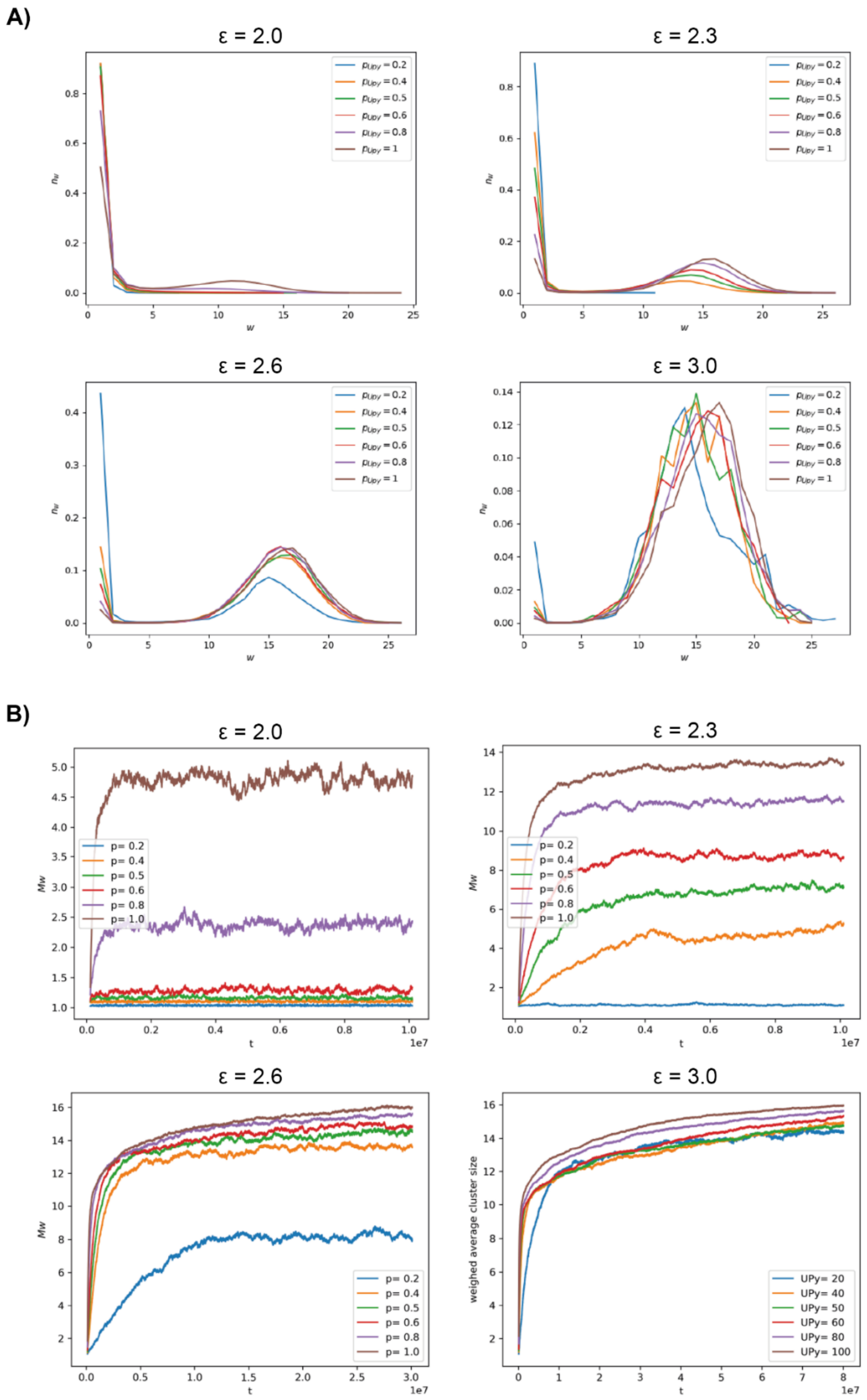

Figure S19. The various UPy molecules have a various degree of clustering. These changes in clustering can be simulated by changing the Lennard-Jones potential between the UPy beads. (A) Cluster distribution of R-cluster size at equilibrium for various interaction strengths (ε=2.0, 2.3, 2.6 and 3.0). The distributions show a clear separation between clustered beads (w > 4) and unbound beads. When the strength of the Lennard-Jones potential increases, the clusters and the amount of beads in a cluster become larger. (B) Weight-averaged cluster sizes over time for various interaction strengths (ε=2.0, 2.3, 2.6 and 3.0). When interaction strength between the UPy's increases it takes longer to reach equilibrium as clusters keep growing.

7. Modulus at various shear strains

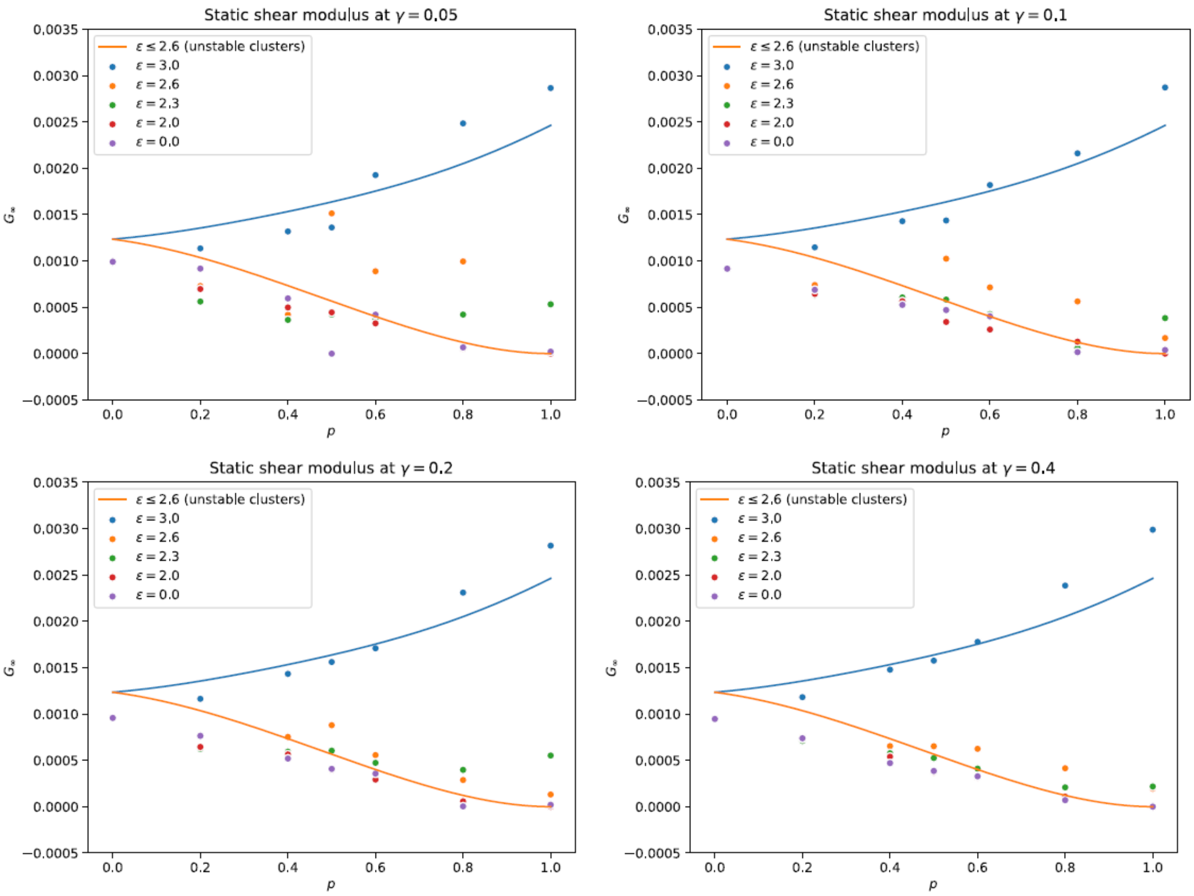

Figure S20. Results of mechanical molecular dynamics simulations, predicting the modulus at various shear strains (γ = 0.05, 0.1, 0.2 and 0.4) and for varying ratio's of incorporated UPy's. Overall trend in the modulus remains similar: Incorporating strong interaction UPy's (∈ = 3.0), the modulus increases when more UPy's are incorporated while for weaker interacting UPy's the modulus decreases with increasing amount of UPy's.

8. Changing the amount of UPy in the network

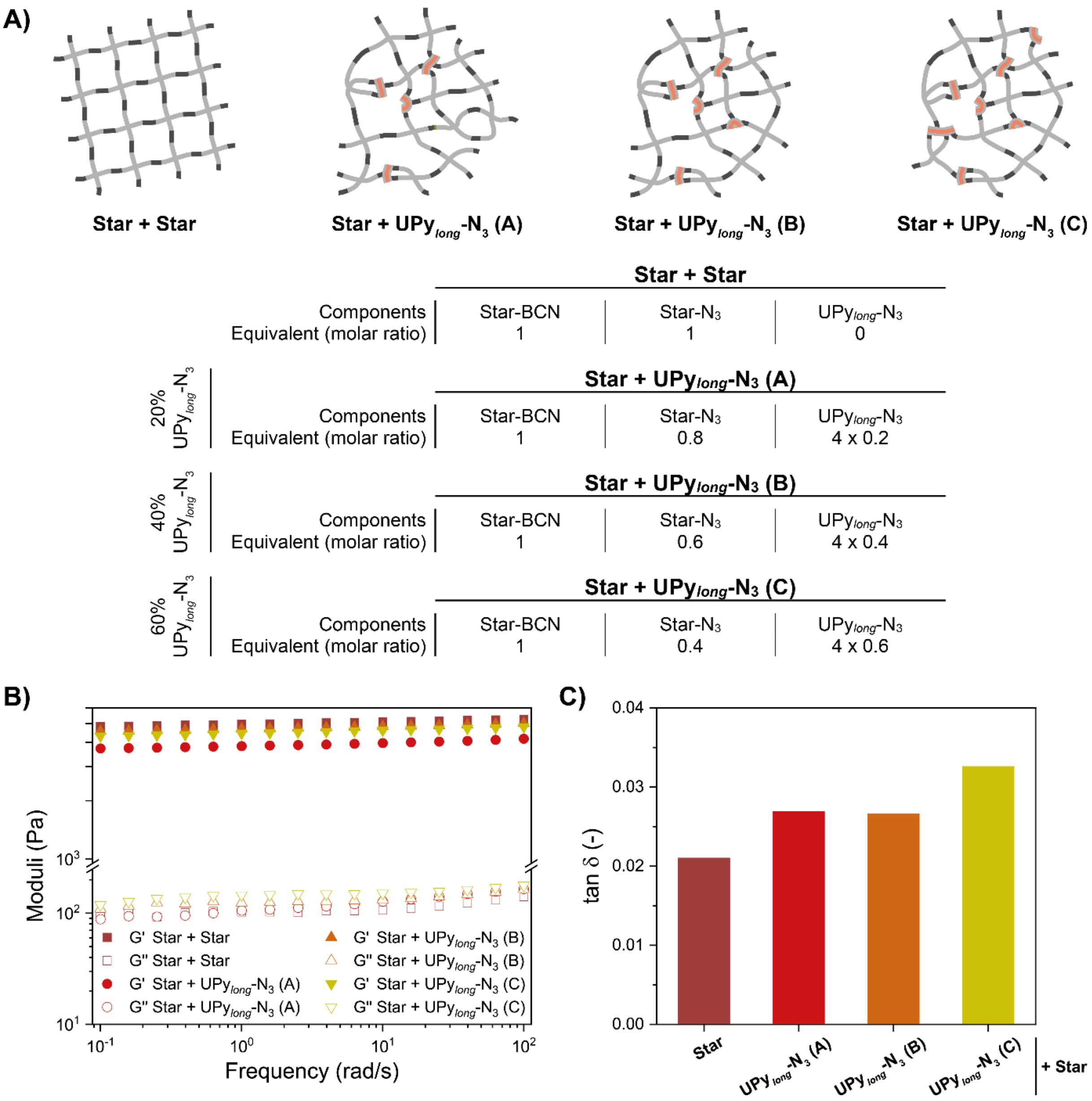

Figure S21. Mechanical comparison of networks composed of various amounts of UPy$_{long}$-N$_3$. (A) Cartoon and table showing molecular composition of hydrogels composed with various amounts of UPy$_{long}$-N$_3$. In a stepwise manner, Star-N$_3$ is replaced by UPy$_{long}$-N$_3$ always making sure there is an equal amount of BCN and N$_3$ moieties. (B) Experimental frequency measurements showing the time dependent mechanical behavior of the different networks (γ=0.01). The results show a marginal difference between the various networks in terms of G' and G''. (C) Effect of different amounts of UPy$_{long}$-N$_3$ on the viscoelastic properties of the hydrogels. Upon incorporation of more UPy$_{long}$-N$_3$, the viscous behavior of the network slightly increased.

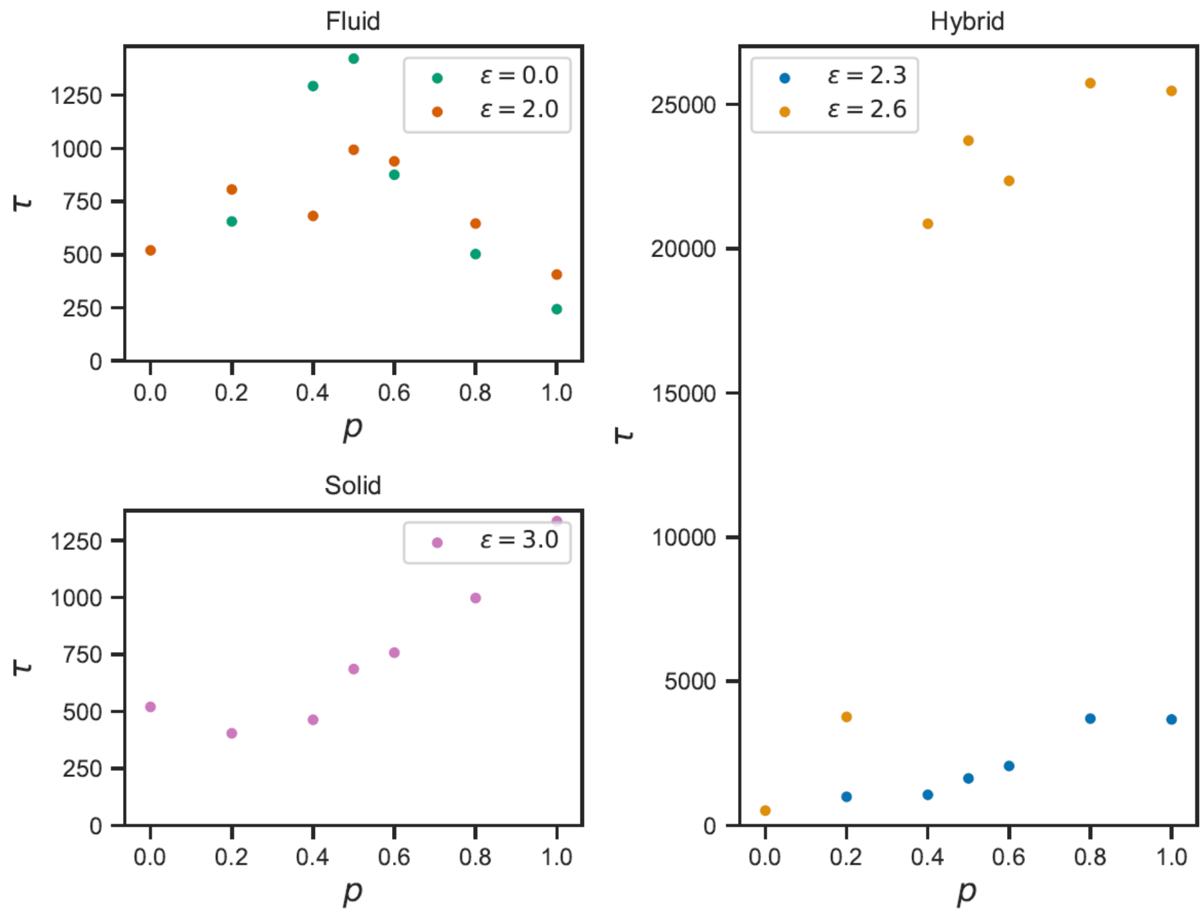

Figure S22. Longest observable relaxation time for varying UPy interaction strength and varying amounts of UPy replacing **Star-N$_3$**. When the interaction is weak (ε = 0 and 2.0) dangling ends are formed around the star molecules. At p = 1 a fluid of single star molecules relaxes very quickly. When p becomes lower the stars can form a percolating network with dangling ends that cause an increase in the relaxation time. When p = 0, a complete covalent network is obtained that relaxes quickly to the plateau modulus. When the interaction strength between the reversible bonds increases (ε = 2.3 and 2.6) the relaxation time increases due to slowly relaxing clusters. In the strongest-binding case (ε = 3.0), the reversible crosslinks do not relax on the time scale of the simulations so the reported timescale is the one governing the relaxation *to* the elastic plateau that the reversible clusters cause. This time scale is much shorter than the stress relaxation time from that plateau to the liquid, which we can only resolve for the smaller values of binding strength.

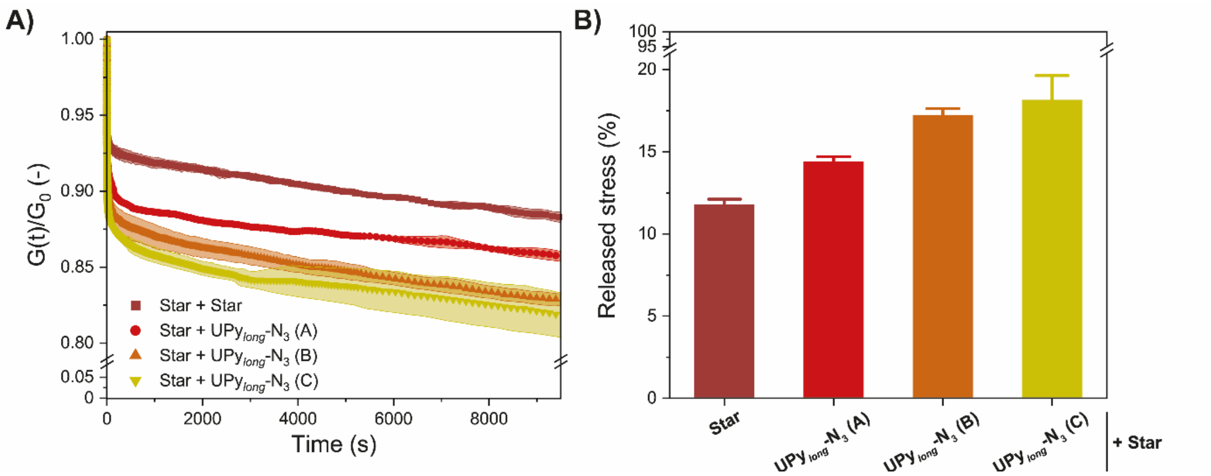

Figure S23. Dynamical properties of hydrogels consisting of varying amounts of UPy_{long}-N_3. (A) Normalized stress relaxation, showing that incorporation of UPy stacks marginally increases the dynamics of the network, i.e. faster stress relaxation. (B) Quantification of the released stress after 10000 seconds.

9. References

1. DeForest, C. A. & Tirrell, D. A. A photoreversible protein-patterning approach for guiding stem cell fate in three-dimensional gels. *Nat. Mater.* **14**, 523–531 (2015).
